# SREBP2-dependent lipid gene transcription enhances the infection of human dendritic cells by Zika virus

**DOI:** 10.1038/s41467-022-33041-1

**Published:** 2022-09-12

**Authors:** Emilie Branche, Ying-Ting Wang, Karla M. Viramontes, Joan M. Valls Cuevas, Jialei Xie, Fernanda Ana-Sosa-Batiz, Norazizah Shafee, Sascha H. Duttke, Rachel E. McMillan, Alex E. Clark, Michael N. Nguyen, Aaron F. Garretson, Jan J. Crames, Nathan J. Spann, Zhe Zhu, Jeremy N. Rich, Deborah H. Spector, Christopher Benner, Sujan Shresta, Aaron F. Carlin

**Affiliations:** 1 Center for Infectious Disease and Vaccine Research, La Jolla Institute for Immunology, La Jolla, CA 92037 USA; 2 Department of Medicine, School of Medicine, University of California, San Diego, La Jolla, CA 92093 USA; 3 School of Molecular Biosciences, College of Veterinary Medicine, Washington State University, Pullman, WA 99163 USA; 4 Biomedical Sciences Graduate Program, University of California, La Jolla, CA 92093 USA; 5 Department of Cellular and Molecular Medicine, School of Medicine, University of California San Diego, La Jolla, CA 92093 USA; 6 Department of Medicine, Division of Regenerative Medicine, University of California San Diego, La Jolla, CA 92093 USA; 7 Sanford Consortium for Regenerative Medicine, La Jolla, CA 92037 USA; 8 Department of Neurology, UPMC Hillman Cancer Center, Pittsburgh, Pennsylvania; Department of Neurology, University of Pittsburgh, Pittsburgh, PA 15232 USA; 9 Department of Pathology, School of Medicine, University of California, San Diego, La Jolla, CA 92093 USA

**Keywords:** Dendritic cells, Pathogens, Systems virology, Virus-host interactions

## Abstract

The emergence of Zika virus (ZIKV) as a global health threat has highlighted the unmet need for ZIKV-specific vaccines and antiviral treatments. ZIKV infects dendritic cells (DC), which have pivotal functions in activating innate and adaptive antiviral responses; however, the mechanisms by which DC function is subverted to establish ZIKV infection are unclear. Here we develop a genomics profiling method that enables discrete analysis of ZIKV-infected versus neighboring, uninfected primary human DCs to increase the sensitivity and specificity with which ZIKV-modulated pathways can be identified. The results show that ZIKV infection specifically increases the expression of genes enriched for lipid metabolism-related functions. ZIKV infection also increases the recruitment of sterol regulatory element-binding protein (SREBP) transcription factors to lipid gene promoters, while pharmacologic inhibition or genetic silencing of SREBP2 suppresses ZIKV infection of DCs. Our data thus identify SREBP2-activated transcription as a mechanism for promoting ZIKV infection amenable to therapeutic targeting.

## Abstract (web summary)

Zika virus (ZIKV) infection suppresses the induction of dendritic cell (DC)-derived immunity, but the underlying mechanistical insights are still lacking. Here the authors show, using in vitro systems profiling of DC transcriptome and epigenome, that ZIKV specifically alters SREBP2-related expression of inflammation- and metabolism-related genes to modulate DC functions.

## Introduction

Zika virus (ZIKV) is an arthropod-borne member of the *Flaviviridae* family of RNA viruses, which includes dengue virus (DENV), West Nile virus, and Japanese encephalitis virus. Outbreaks of ZIKV in many parts of the world, including the Americas, led the World Health Organization to declare ZIKV a public health emergency of global concern in 2016. Although most ZIKV infections cause short-lived and mild symptoms, they can also lead to severe complications with devastating consequences, particularly Guillain–Barré syndrome in adults^1,2^ and congenital Zika syndrome in infants born to ZIKV-infected mothers^3–8^. Despite intense research on ZIKV pathogenesis, the mechanisms by which the virus productively infects target cells remain unclear.

Dendritic cells (DCs) play crucial roles in detecting viral pathogens and orchestrating short- and long-term antiviral responses through both the innate and adaptive immune systems. DCs exist as multiple subsets, including plasmacytoid DCs, classical DCs, and monocyte-derived DCs (moDCs), with diverse ontogeny, phenotypes, and functions. Upon viral infection, DCs activate vigorous antiviral responses focused on the production of and response to type I interferons (IFNs), and they then undergo maturation to become potent antigen-presenting cells for virus-specific T cells. However, many viruses have evolved mechanisms to subvert DC function, thereby suppressing host defenses.

ZIKV can infect multiple DC subsets, including moDCs^9–11^. Analysis of ZIKV-infected moDCs and primary myeloid DCs from infected individuals have shown that ZIKV suppresses the antiviral response, including production of type I IFN, as well as DC maturation and activation^9,12^. However, little is known about the molecular mechanisms and pathways by which ZIKV enables productive infection of DCs. Previous studies have analyzed the transcriptional programs activated in cells infected with ZIKV, but it has proven difficult to identify transcription factors (TFs) responsible for changes in gene expression^13–16^. Therefore, in the present study, we employed a highly sensitive approach recently developed by our laboratory to deconvolute the genomic profiles of purified ZIKV-infected and bystander uninfected cells, thereby facilitating the identification of key regulatory TFs that control DC responses specifically during ZIKV infection^17–19^.

Here we identify transcriptional programs regulated by ZIKV by comparing genome-wide transcriptional profiles of highly purified ZIKV-infected and uninfected bystander moDCs with mock-infected cells. Notably, ZIKV infection is associated with increased expression of genes enriched in lipid metabolism-related functions. Chromatin immunoprecipitation sequencing (ChIP-seq) analyses reveals that sterol regulatory element-binding proteins (SREBPs), the master regulatory TFs of lipid metabolism, are preferentially recruited to the promoters of lipid metabolism-related genes, and capped small RNA-seq (csRNA-seq) analysis demonstrates increased transcription initiation of these genes. Further mechanistic investigation demonstrates that SREBP2 activity promotes ZIKV infection of moDCs. These findings identify a novel mechanism by which ZIKV creates a favorable environment for replication in moDCs and also suggest that SREBP2-dependent lipid metabolism is a potential therapeutic target to suppress ZIKV infection.

## Results

### ZIKV productively infects human moDCs

To investigate ZIKV–moDC interactions that determine the outcome of infection, we established a moDC model in which primary human monocytes were differentiated to moDCs in culture and then infected for various lengths of times with the Asian lineage ZIKV strain SD001^17^ at a multiplicity of infection (MOI) of 0.5. Viral infection was monitored by flow cytometry of cells stained with 4G2, a pan flavivirus envelope (E) protein-specific monoclonal antibody (mAb). The mean infectivity rates at 6, 12, 18, 24, and 48 h post-infection (pi) were 0.3%, 17.1%, 34.7%, 43.5%, and 23.2%, respectively (Supplementary Fig. 1a). A single population of 4G2+ cells was observed up to 48 h pi, consistent with a single round infection without spread to uninfected bystanders (Supplementary Fig. 1b). Infectious viral particles in the culture supernatants, as measured using cell-based focus-forming assays (FFAs), increased progressively from 6 h until 24 h pi, after which the same level of infection was observed until ≥48 h pi (Supplementary Fig. 1c). Thus, moDCs support productive ZIKV infection with a peak infection time of 24 h pi.

### ZIKV infection reprograms expression of lipid metabolism-related genes in moDCs

**Fig. 1:**
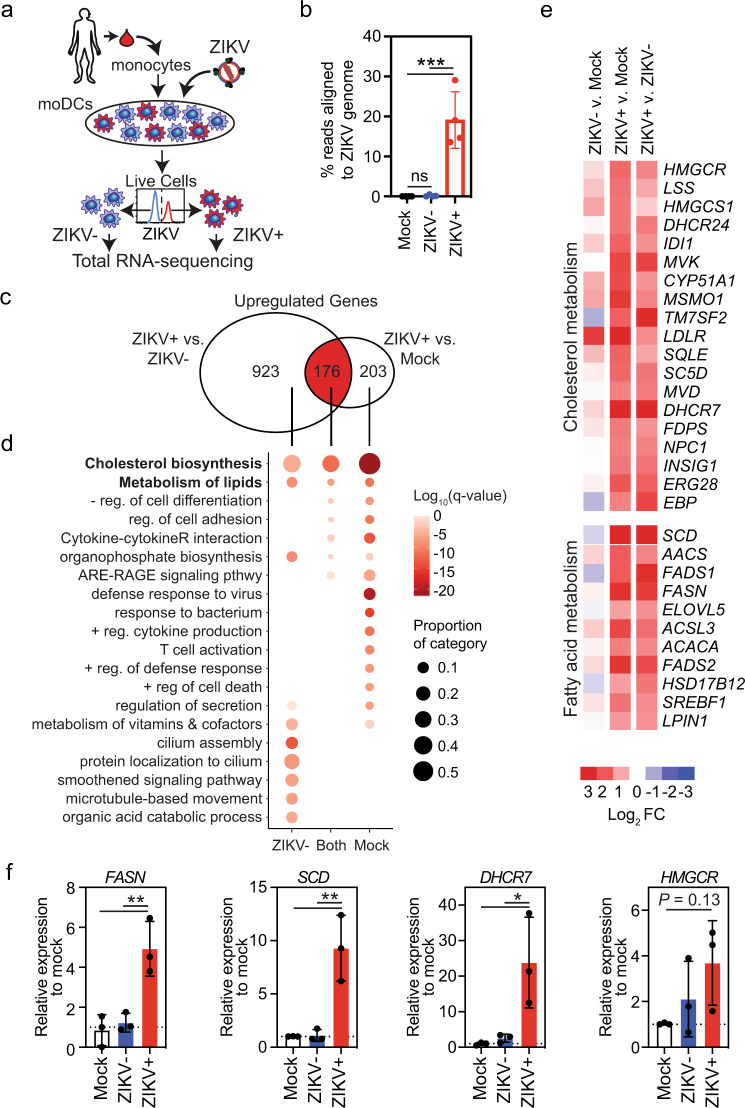
ZIKV infection of human moDCs reprograms expression of lipid-related genes. **a** Human moDCs from four different donors were infected for 24 h with ZIKV SD001 at MOI 0.5. Cells were then stained for the viral envelope (E) protein using mAb 4G2 and sorted into ZIKV-infected (ZIKV+) and bystander uninfected (ZIKV−) cells. Total RNA was isolated and subjected to RNA-seq. Mock-infected cells (Mock) were analyzed in parallel. **b** Percent of reads aligned to the ZIKV genome in each cell population. **c** Venn diagram showing the number of unique and shared genes upregulated (fold-change >2, false discovery rate <0.01) in ZIKV+ vs ZIKV− or Mock moDCs. **d** Gene ontology analysis of genes significantly upregulated in ZIKV+ vs ZIKV− or Mock moDCs. **e** Heat map of the relative expression of selected genes implicated in lipid metabolism. **f** qRT-PCR analysis of the relative expression of fatty acid (*FASN* and *SCD*) and cholesterol (*DHCR7* and *HMGCR*) synthesis genes. Data are presented as the mean ± SD. *n* = 4 (**b**) and *n* = 3 (**f**) biologically independent experiments. Symbols represent moDCs derived from individual donors. *P < 0.05, **P < 0.01, ***P < 0.001 by one-way ANOVA with Tukey’s correction for multiple comparisons. Source data and exact *P* values are provided as a Source Data file.

To detect host genes specifically regulated by ZIKV, we infected moDCs derived from 4 donors with ZIKV SD001 (MOI 0.5) for 24 h and then separated cells from the same culture into ZIKV-infected (ZIKV+) and ZIKV-exposed but uninfected bystander (ZIKV−) moDCs by staining with 4G2 followed by fluorescence-activated cell sorting (FACS) (Fig. 1a)^17^. Mock-infected cells (referred to as Mock moDCs) from each donor were included in each assay. The cells were analyzed by ribosomal-depleted strand-specific RNA-seq, and the RNA-seq reads were aligned to a combined human (GRCh38/hg38) and ZIKV genome. Only the ZIKV+ population had significant reads aligning to the ZIKV genome (Fig. 1b), confirming the efficiency of the FACS method for separation of ZIKV+ and ZIKV− cell populations.

We identified a total of 1099 and 379 genes that were upregulated to a greater extent (fold-change [FC] > 2 and false discovery rate [FDR] < 0.01) in ZIKV+ moDCs compared with ZIKV− or Mock moDCs, respectively. Of these genes, 176 were upregulated in ZIKV+ cells compared with both ZIKV− and Mock moDCs (Fig. 1c), indicating that they were specifically induced by ZIKV infection. The 1099 and 379 genes upregulated in ZIKV+ vs. ZIKV− or Mock cells, respectively, were enriched for gene ontology (GO) and pathway terms related to lipid synthesis, viral defense, and immune responses (Fig. 1d). The 176 genes exclusively upregulated in ZIKV+ cells were also enriched for lipid synthesis pathway terms but not for most viral response or immune response categories (Fig. 1d); indeed, expression of the latter two gene sets were largely suppressed in ZIKV+ compared with ZIKV− moDCs (Supplementary Fig. 2a). These findings suggest that ZIKV actively suppresses the synthesis of immune response-related genes in infected moDCs. Although type I and III IFN genes were most strongly upregulated in ZIKV+ moDCs, interferon-stimulated gene (ISG) expression was suppressed in ZIKV+ compared with ZIKV− moDCs (Supplementary Fig. 2a). Thus, although infected moDCs are capable of upregulating IFN expression, they cannot respond to the secreted IFNs, which is consistent with the known ability of ZIKV to suppress IFN signaling in human cells by targeting the TF signal transducer and activator of transcription protein 2 (STAT2)^20^.

The relatively small number of genes specifically induced by ZIKV infection (Supplementary Fig. 2b) included a high frequency of uniformly upregulated genes related to lipid metabolism, including cholesterol and fatty acid biosynthesis-related genes (Fig. 1e). We validated the RNA-seq results by qRT-PCR analysis of two fatty acid biosynthetic genes, fatty acid synthase (*FASN)* and stearoyl-CoA desaturase (*SCD*), and two cholesterol biosynthetic genes, 7-dehydrocholesterol reductase (*DHCR7)* and HMG-CoA reductase (*HMGCR*), which were markedly upregulated in ZIKV+ moDCs compared with either ZIKV− or Mock moDCs (Fig. 1f). We determined the functional outcome of these transcriptional changes by quantifying lipid levels in moDCs by intracellular staining with the lipid-binding fluorescent dye BODIPY 493/503 followed by flow cytometry, revealing higher accumulation of neutral lipids in ZIKV+ compared with ZIKV− and Mock moDCs (Supplementary Fig. 2c). Taken together, these data demonstrate that ZIKV infection of moDCs induces expression of a large number of cholesterol and fatty acid metabolism-related genes and results in an increase in lipid stores.

### Reprogramming of lipid metabolism-related genes is cell type- and virus-specific

**Fig. 2:**
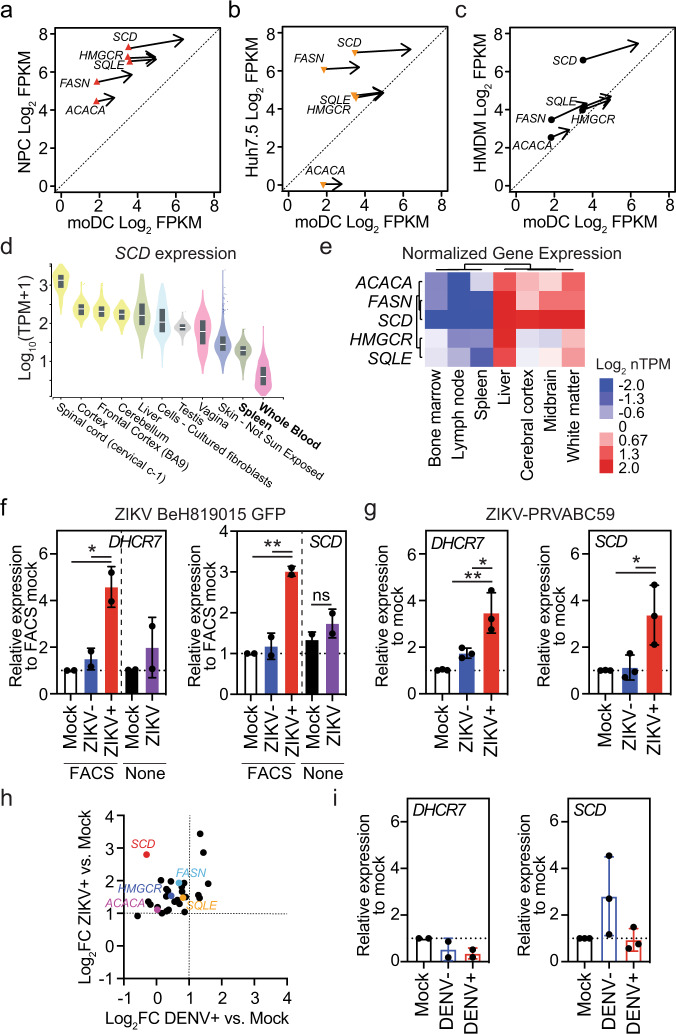
Cell type- and virus-specific reprogramming of lipid metabolism genes. **a**–**c** Effect of ZIKV infection on the expression of rate-limiting enzymes in cholesterol and fatty acid synthesis in **a** neural progenitor cells (NPCs), **b** Huh7.5 human hepatoma cells, and **c** human monocyte-derived macrophages (HMDMs) compared with moDCs. Each symbol represents the baseline expression and the arrows point to the expression in ZIKV+ cells. **d** *SCD* expression in tissues that can be infected by ZIKV in order of median expression level (data from GTEx Analysis Release V8). The box shows the median value and extends from the 25th to 75th percentile, while the whiskers extend from min to max values. Points are displayed as outliers if they are above or below 1.5 times the interquartile range. **e** Unsupervised clustering of normalized expression (Log_2_ nTPM) of the indicated genes in the indicated tissues (data from The Human Protein Atlas v21.0)^22^. **f**, **g** qRT-PCR analysis of the relative expression of *DHCR7* and *SCD* in moDCs infected for 24 h with **f** ZIKV BEH819015 (MOI 1) or **g** ZIKV PRVABC59 (MOI 1). **h** RNA-seq analysis of the relative expression of lipid biosynthetic genes (listed in Fig. 1e) in ZIKV+ (SD001) and DENV2+ (UIS353) moDCs vs Mock moDCs at 24 h post-infection. Enzymes catalyzing rate-limiting steps in cholesterol and fatty acid synthesis are highlighted. **i** qRT-PCR analysis of the relative expression of *DHCR7* and *SCD* in moDCs infected for 24 h with DENV2 (UIS353) at MOI 1. Data are presented as the mean ± SD. Symbols represent moDCs derived from individual donors *n* = 2 (**f**) and *DHCR7* (**i**) or *n* = 3 (**g**) and *SCD* (**i**) biologically independent experiments. *P < 0.05, **P < 0.01 by one-way ANOVA with Tukey’s correction for multiple comparisons (**g**), (**i**) and (**f**) [FACS] or two-sided unpaired *t* test (**f**) [None]. Source data and exact *P* values are provided as a Source Data file.

ZIKV can infect many immune and non-immune human cell types, including neural progenitor cells (NPCs), hepatocytes, and macrophage^21^. We previously showed that genes involved in lipid synthesis are broadly upregulated in ZIKV+ compared with ZIKV− and mock-infected human monocyte-derived macrophage (HMDMs) (Supplementary Fig. 3a)^17^. To determine whether lipid metabolism genes are also regulated by ZIKV infection of other cell types, we employed the same approach that we used for moDCs to infect, isolate, and perform RNA-seq on human pluripotent stem cell-derived NPCs and Huh7.5 cells, a human hepatoma cell line. We first compared the expression of the key rate-limiting enzymes in cholesterol and fatty acid synthesis in ZIKV+ and ZIKV− moDCs with the equivalent populations of NPCs, Huh7.5 cells, and HMDMs. Compared with the observation in moDCs and HMDMs, ZIKV infection had a smaller effect on upregulation of lipid synthesis genes in NPCs and had little-to-no effect in Huh7.5 cells (Fig. 2a–c). Moreover, the baseline expression level of lipid genes in uninfected NPCs and Huh7.5 cells was much higher than in the two innate immune cell types (Supplementary Fig. 3b). Investigation of the expression of *SCD* in human tissues through the Genotype-Tissue Expression Project (gtexportal.org) showed that *SCD* was expressed at the lowest levels in immune organs (spleen and blood) compared with the other tissues analyzed (Fig. 2d). These findings were confirmed by clustering of normalized expression of Acetyl-CoA Carboxylase Alpha (*ACACA), FASN, SCD, HMGCR*, and Squalene Epoxidase (*SQLE)* (Human Protein Atlas; proteinatlas.org), which demonstrated lower expression of these genes in immune tissues (bone marrow, lymph nodes, spleen) and higher expression in brain and liver (Fig. 2e)^22^. Notably, although lipid gene expression was high in liver and brain, resident immune cells in these tissues, such as Kupffer cells and microglia, respectively, expressed lower levels of *HMGCR* and *FASN* than did other cell types from those tissues (e.g., hepatocytes, neurons) (Supplementary Fig. 3c). Thus, ZIKV infection regulates the expression of lipid-related genes in a cell type-specific manner, and upregulation of lipid-related genes may be of particular importance to ZIKV infection of immune cells, in which baseline expression of these genes is low.

We next investigated whether regulation of lipid metabolism genes in moDCs is ZIKV strain-specific by examining cells infected with ZIKV-EGFP-BeH819015 or ZIKV PRVABC59^23,24^. qRT-PCR analysis showed that *DHCR7*, *SCD*, and *HMGCR* mRNA levels were significantly increased in moDCs infected with ZIKV-EGFP-BeH819015 (Fig. 2f) or ZIKV PRVABC59 (Fig. 2g) compared with Mock and ZIKV− moDCs, as was observed following infection with ZIKV SD001. Analysis of unsorted moDCs containing a mixture of ZIKV+ and ZIKV− cells did not reveal significant upregulation of these genes (Fig. 2f and Supplementary Fig. 3d). These data demonstrate that lipid metabolism-related genes are induced by infection of moDCs with multiple Asian lineage strains of ZIKV. Further, they highlight the importance of analyzing isolated ZIKV+ cells to increase the sensitivity of detection of virally regulated host genes.

To determine if induction of lipid gene upregulation in moDCs by ZIKV was specific or shared with other closely related flaviviruses, we performed similar experiments with moDCs infected with a DENV serotype 2 (DENV2) clinical strain UIS353. Similar to ZIKV, DENV2 infection of moDCs peaked at 24 h pi and induced secretion of infectious viruses, as assessed by flow cytometry of 4G2+ cells and FFA of culture supernatants (Supplementary Fig. 3e, f). Indeed, ZIKV and DENV2 infection of moDCs derived from up to 6 donors revealed no significant differences in either percent infection or infectious virus secretion at any time point (Supplementary Fig. 3e–g). Despite similar efficiencies of ZIKV and DENV to infect moDCs, DENV infection failed to upregulate the expression of most lipid biosynthesis-related genes, as detected by RNA-seq of *ACACA*, *FASN*, *SCD*, *HMGCR*, and *SQLE* in DENV+, DENV−, and Mock moDCs at 24 h pi (Fig. 2h). These results were confirmed by qRT-PCR analysis of *SCD* and *DHCR7* mRNA levels (Fig. 2i). Collectively, these results indicate that lipid-related gene expression in moDCs is stimulated by several strains of Asian lineage ZIKV, but not by DENV2 UIS353, suggesting that this mechanism may be of particular importance for ZIKV infection of immune cells.

### SREBP TFs control lipid-related gene transcription in ZIKV-infected moDCs

**Fig. 3:**
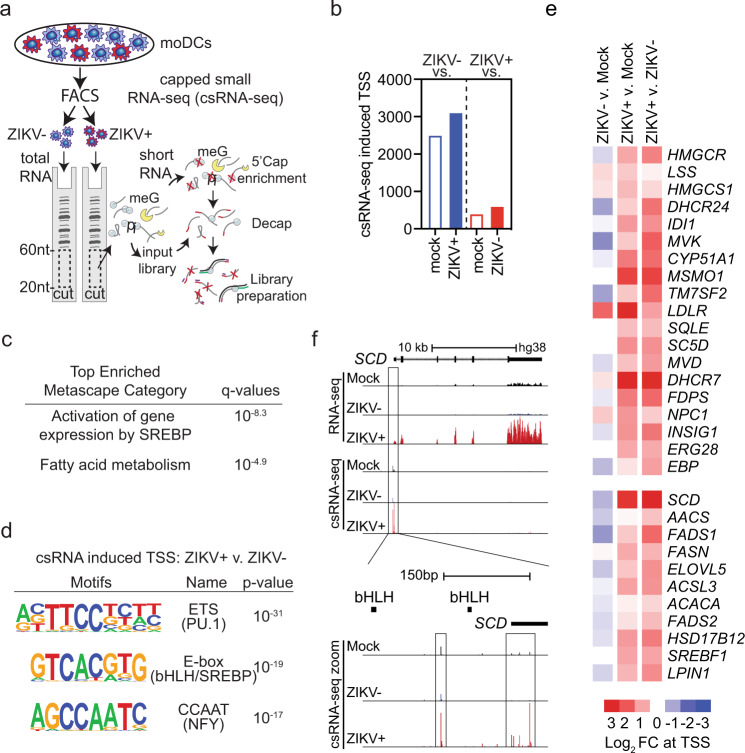
ZIKV infection of human moDCs induces de novo expression of lipid-related genes. **a** moDCs from 3 donors were infected for 24 h with ZIKV SD001 (MOI 0.5) and cells were FACS sorted into ZIKV− and ZIKV+ populations. RNA from Mock, ZIKV−, and ZIKV+ moDCs were subjected to csRNA-seq. **b** Number of upregulated (fold-change >2, false discovery rate <0.01) TSRs. **c** Most enriched Metascape pathways associated with genes containing upregulated promoter proximal TSRs in ZIKV+ moDCs vs ZIKV− or Mock moDCs. **d** De novo motif analysis of TSRs upregulated in ZIKV+ moDCs vs ZIKV− moDCs. **e** Heat map of csRNA-seq reads at the TSRs of selected genes implicated in the lipid metabolism. **f** UCSC browser visualization of the *SCD* locus with RNA-seq and csRNA-seq in Mock, ZIKV−, and ZIKV+ moDCs. RNA-seq and csRNA-seq are strand-specific, with positive- and negative-strand transcription displayed above and below the central line, respectively.

We next sought to identify TFs involved in the induction of lipid metabolism genes in ZIKV-infected moDCs by using csRNA-seq^19^, a highly sensitive method for analysis of gene regulatory networks that quantifies changes in transcription initiation at both promoters and distal regulatory elements^19^. In contrast to analysis of mRNA levels, which may be altered by changes in synthesis, processing, or degradation, csRNA-seq captures active transcription initiation events occurring at the time of analysis, similar to methods that quantify nascent RNAs^25^. However, unlike the latter methods, csRNA-seq requires only total RNA, not intact nuclei, and is therefore compatible with cell fixation. To identify genes and distal regulatory elements undergoing active transcription in fixed ZIKV+, ZIKV−, and Mock moDCs (Fig. 3a), we performed csRNA-seq and identified ~76,701 transcription start regions (TSRs) comprised of one or more closely spaced transcription start sites (TSSs). Of these TSRs, ~37% were in promoters and 63% were in distal regulatory elements. As expected, only the ZIKV+ moDCs contained csRNA-seq reads aligning to the ZIKV genome (Supplementary Fig. 4a). ZIKV− moDCs contained 2494 and 3096 upregulated (FC > 2, FDR < 0.01) TSRs compared with Mock or ZIKV+ moDCs, respectively (Fig. 3b). In comparison, ZIKV+ moDCs contained far fewer upregulated TSRs: 395 compared with Mock and 595 compared with ZIKV− moDCs, and of these, 121 were overlapping (Fig. 3b). TSRs in the promoter proximal region (−500 bp to +500 bp from GRCh38/hg38 annotated TSS) are more reliably linked to the expression of a specific gene than are distal TSRs. TSRs in the promoter proximal regions of 48 genes were upregulated in ZIKV+ compared with both ZIKV− and Mock moDCs. These genes were highly enriched for pathways related to SREBP-activated gene expression and fatty acid metabolism (Fig. 3c), which is consistent with the crucial role played by SREBPs in regulating the expression of genes involved in lipid biosynthesis^26^. De novo motif analysis demonstrated that TSSs specifically induced by ZIKV infection were enriched for ETS, enhancer box (E-box), and CCAAT motifs (Fig. 3d). ETS motifs can be bound by PU.1, a lineage-determining TF that positively regulates genes in myeloid lineages; SREBPs are basic helix–loop–helix leucine zipper (bHLH-LZ) TFs that can bind to E-box and sterol regulatory element (SRE) motifs (Fig. 3d)^27,28^; and the CCAAT motif can be bound by Nuclear Transcription Factor Y (NFY), a TF known to cooperate with SREBPs to activate transcription (Fig. 3d)^29–31^. Upregulation of promoter proximal TSRs occurred broadly across lipid biosynthetic genes and was generally specific to ZIKV+ cells (Fig. 3e). RNA-seq and csRNA-seq demonstrated increased read density over *SCD* and *HMGCR* exons and TSSs, respectively (Fig. 3f and Supplementary Fig. 4b). This suggests that the increases in lipid gene transcripts observed in ZIKV+ moDC are at least partly due to upregulation of nascent transcription. Increased TSSs in TSRs associated with the *SCD* and *HMGCR* promoter proximal region were located directly downstream, ~50–200 bp, from E-box motifs, which suggests that TF binding to these motifs contributes to the initiation of transcription at these sites (Fig. 3f and Supplementary Fig. 4b). These results support the findings detected by RNA-seq analysis and indicate that ZIKV infection of moDCs induces transcription at only a small number of promoters and enhancers (Supplementary Figs. 2b and 3b), and that the response is highly enriched for genes involved in lipid synthesis and involves TF motifs implicated in SREBP-dependent regulation (Fig. 3c–e).

In addition to SREBP TFs, X-Box Binding Protein 1 (XBP1) induces transcription of selected lipid metabolism-related genes in response to endoplasmic reticulum (ER) stress^32^. During ER stress, the endoribonuclease domain of inositol-requiring enzyme 1α (IRE1α) initiates nonconventional cytoplasmic splicing of unspliced *XBP1* (u*XBP1*) to spliced *XBP1* (s*XBP1*), which is then translated into the active XBP1 TF^33^. XBP1 binds ER stress response element (ERSE) or unfolded protein response element (UPRE) motifs to induce expression of stress response genes^32^. Although we did not identify upregulated ERSE or UPRE motifs in ZIKV+ moDCs by csRNA-seq, previous studies have shown that ZIKV can promote infection by activating IRE1α and XBP1^34,35^. To determine if this occurs during ZIKV infection of moDCs, we performed qRT-PCR to quantify active s*XBP1* and inactive u*XBP1* in Mock, ZIKV− and ZIKV+ moDCs at 24 h pi. ZIKV infection did not alter *XBP1* splicing in moDCs, while treatment with the ER stress inducer tunicamycin increased and decreased s*XBP1* and u*XBP1* mRNA levels, respectively, as expected (Supplementary Fig. 4c,d). In response to ER stress, XBP1, ATF6, and ATF4 TFs induce expression of genes involved in ER stress resolution (*PPP1R15A*), ER-associated degradation (*EDEM1*, *HERPUD1*), as well as ER chaperones (*HSPA5*, *DNAJB9*, *PDIA3*, *DNAJB11*) in addition to *XBP1*^32^. ER stress genes were upregulated in moDCs in response to infection with DENV2, but not ZIKV (Supplementary Fig. 4e), identifying another difference in the outcomes of infection of moDCs by these flaviviruses. As ZIKV infection did not induce *XBP1* splicing, transcription initiation at ERSE or UPRE motifs, or expression of ER stress response genes XBP1 is unlikely to be responsible for the observed induction of lipid metabolism genes in response to ZIKV infection. Thus, SREBPs are the TFs most likely to upregulate the expression of lipid metabolism-related genes in ZIKV-infected moDCs.

### ZIKV infection increases SREBP recruitment to upregulated lipid metabolism genes

**Fig. 4:**
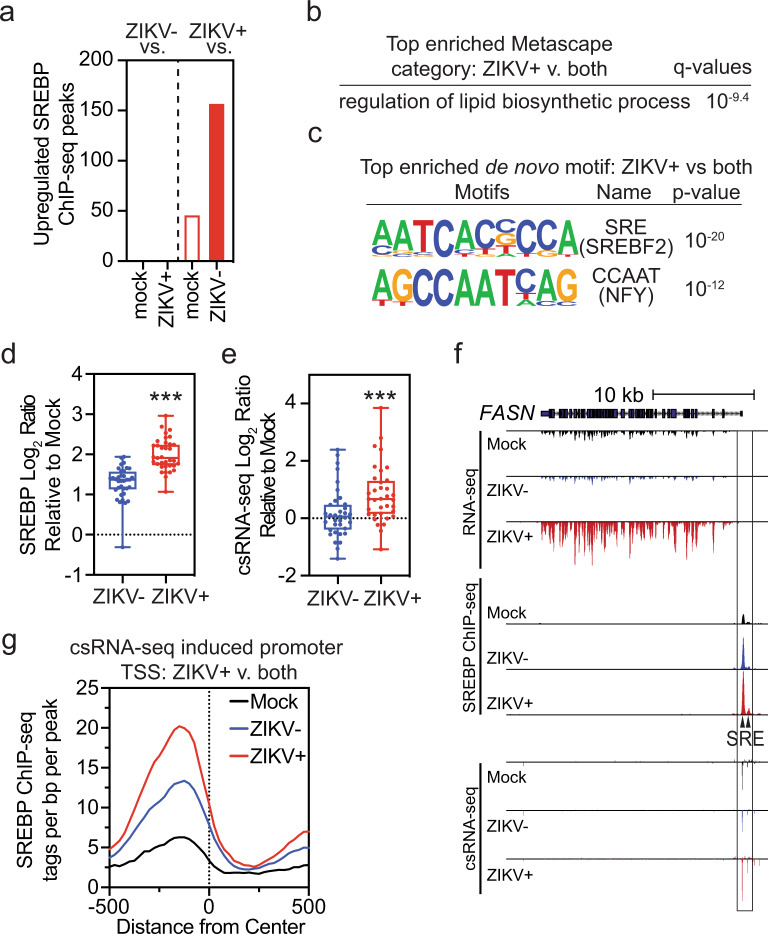
ZIKV infection of human moDCs increases SREBP recruitment and transcription of lipid-related genes. **a**–**g** ChIP-seq analysis was performed to detect SREBP TF binding to DNA in Mock, ZIKV−, or ZIKV+ moDCs from 3 donors infected for 24 h with ZIKV PRVABC59 (MOI 1). **a** Number of upregulated (fold-change >1.5, false discovery rate <0.1) SREBP peaks. **b** Most enriched Metascape pathways among genes with increased SREBP binding in ZIKV+ vs ZIKV− and Mock moDCs. **c** De novo motif analysis of upregulated SREBP peaks in ZIKV+ vs ZIKV− and Mock moDCs. **d**, **e** Log_2_ ratio of **d** SREBP or **e** csRNA-seq tags from −500 bp to +500 bp at upregulated SREBP peaks in ZIKV+ or ZIKV− vs Mock moDCs. All points representing upregulated ZIKV+ SREBP peaks are shown. Box plots show the median value and extend from the 25th to 75th percentile, while the whiskers extend from min to max values. *N* = 3 biologically independent experiments. ***P < 0.001 by two-sided paired *t* test. **f** UCSC browser visualization of the *FASN* locus with RNA-seq, SREBP ChIP-seq, and csRNA-seq in Mock, ZIKV−, and ZIKV+ moDCs. Location of SREBP (SRE) motifs is indicated. **g** SREBP-binding relative to csRNA-seq promoter proximal TSS upregulated in ZIKV+ vs ZIKV− and Mock moDCs. Source data and exact *P* values are provided as a Source Data file.

To determine whether or not ZIKV infection activates SREBP TFs in moDCs, we performed ChIP-seq on Mock, ZIKV+, and ZIKV− cells. A total of 16,800 SREBP-binding sites were identified, of which 187 were specifically upregulated (FC > 1.5, FDR < 0.1) in ZIKV+ compared with ZIKV− or Mock moDCs. Of these, 35 were upregulated in ZIKV+ compared with both ZIKV− and Mock cells, whereas no SREBP-binding sites were similarly increased in ZIKV− cells (Fig. 4a). The 35 genes identified as specific to ZIKV+ cells were most strongly associated with lipid biosynthetic processes (Fig. 4b), and de novo motif analysis of these sites showed specific enrichment for SRE motifs (bound by SREBP TFs) and CCAAT binding motifs (bound by NFY, which cooperates with SREBPs) (Fig. 4c). Analysis of upregulated peaks showed greater increases in SREBP binding and local transcription initiation in ZIKV+ cells compared with ZIKV− moDCs (Fig. 4d, e). SREBP binding was increased in ZIKV+ moDCs at the promoters of *FASN*, *LDLR*, and *DHCR7* genes overlying SRE motifs and was associated with increased csRNA-seq TSR reads immediately downstream of SREBP binding (Fig. 4f and Supplementary Fig. 5). Analysis of all promoter proximal csRNA-seq TSRs upregulated specifically in ZIKV+ moDCs demonstrated increased SREBP binding immediately upstream of the TSR (Fig. 4g). Taken together, ZIKV infection increases SREBP binding at lipid metabolism-related genes and SREBP binding is associated with increased downstream transcription initiation, consistent with activation of these promoters.

### Inhibition of SREBP2 reduces productive ZIKV infection of moDCs

**Fig. 5:**
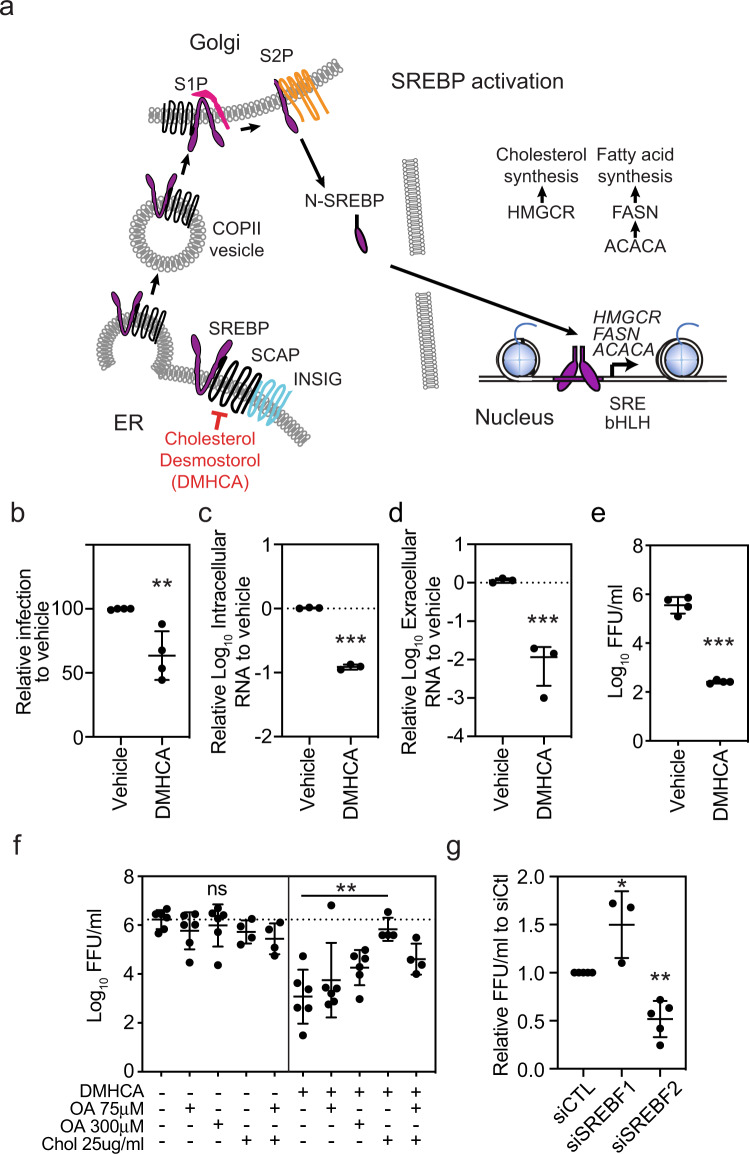
Treatment of moDCs with the SREBP2 inhibitor DMHCA suppresses ZIKV infection. **a** Simplified model of the SREBP activation pathway. **b** moDCs were treated with vehicle (ethanol) or DMHCA (10 μM) for 4 h, infected with ZIKV PRVABC49 (MOI 1) for 24 h, and ZIKV-infected cells were quantified by 4G2 staining and flow cytometry. **c**–**e** moDCs were infected with ZIKV PRVABC49 (MOI 1) and treated with DMHCA (10 μM) at 2.5 h post infection (pi). At 24 h pi, **c** intracellular and **d** extracellular ZIKV RNA were quantified by qRT-PCR and **e** infectious virions were quantitated by focus-forming assay (FFA). Data are presented as the mean ± SD. *n* = 4 (**b**, **e**) or *n* = 3 (**c**, **d**) biologically independent experiments. **f** moDCs were infected with ZIKV PRVABC59 (MOI 1) for 1 h, washed, and treated with vehicle (left) or DMHCA 10 μM (right) in the presence or absence of oleic acid–BSA (OA) and/or cholesterol–methyl-β-cyclodextrin (Chol) at the indicated concentrations. At 24 h pi, infectious virions were quantitated by FFA. Data are presented as the mean ± SD. *n* = 4, for Chol treatment, or *n* = 6, for all other treatments, biologically independent experiments. **g** moDCs were transfected with siRNAs targeting SREBF1 or SREBF2 for 24 h before infection with ZIKV SD001 (MOI 0.5). At 24 h pi, infectious virions in the supernatants were quantified by FFA. Data are presented as the mean ± SD. *n* = 5 (siCTL, siSREBF2) or *n* = 3 (siSREBF1) biologically independent experiments. Symbols represent moDCs derived from individual donors. **P < 0.01, ***P < 0.001 by two-sided unpaired *t* test (**b**–**e**) or one-way ANOVA with Dunnett’s correction for multiple comparisons (**f**, **g**). Source data and exact *P* values are provided as a Source Data file.

Membrane-bound SREBP precursor proteins form a complex with SREBP cleavage-activating protein (SCAP) in the ER (Fig. 5a)^36^. SCAP binds reversibly to insulin induced gene (INSIG)−1 or INSIG-2 in the presence of sterols, thereby retaining the inactive form of SREBPs in the ER^37^. When INSIG–SCAP interactions are disrupted, the SREBP–SCAP complex is transported in COPII vesicles to the Golgi, where SREBPs are proteolytic cleaved by site-1 and site-2 proteases. Proteolytic cleavage releases N-terminal SREBP fragments, which dimerize and bind to specific DNA sequences such as SRE or E-box motifs found near the promoters of enzymes involved in cholesterol and fatty acid biosynthesis^38^. SREBP1a, the longer isoform of SREBP1, can induce all SREBP-responsive genes, whereas SREBP2 preferentially activates genes involved in cholesterol synthesis^39^.

As ZIKV infection specifically increased SREBP recruitment and nascent transcription of lipid synthesis genes in moDCs, we hypothesized that inhibition of SREBP TFs would suppress infection. To test this hypothesis, we evaluated the effects on ZIKV infection of N,N-dimethyl-3β-hydroxycholenamide (DMHCA), a selective liver X receptor (LXR) modulator that has a sterol-like structure and suppresses SREBP processing, most likely by binding to SCAP^40,41^. Incubation of uninfected moDCs with DMHCA did not significantly affect cell viability or metabolism (Supplementary Fig. 6a), confirming that it is not cytotoxic. Preincubation of moDCs with DMHCA 4 h prior to infection with ZIKV PRVABC59 or SD001 inhibited infection by 36% and 47%, respectively (Fig. 5b and Supplementary Fig. 6b). DMHCA treatment also reduced the level of intracellular virus RNA by 8-fold, extracellular virus RNA by 86-fold, and the number of secreted infectious ZIKV PRVABC59 and SD001 particles by >1000-fold and 257-fold, respectively (Fig. 5c–e and Supplementary Fig. 6c). Comparing the effects of DMHCA when added 4 h before or 2.5 h after ZIKV infection, this inhibitor primarily blocks infection post-entry (Supplementary Fig. 6d, e). Although DMHCA could inhibit virus replication, assembly, budding, and/or egress, quantification of intracellular and extracellular infectious virus particles at 24 h pi showed no significant difference between DMHCA- and vehicle-treated moDCs (Supplementary Fig. 6f), suggesting that DMHCA does not affect secretion of viral particles. Taken together, DMHCA inhibits ZIKV post-entry, most likely by suppressing replication, assembly, and/or budding, rather than egress of infectious particles.

We next examined whether DMHCA might inhibit ZIKV infection in moDCs by interfering with the production of cholesterol and/or fatty acids. SCD1 catalyzes the rate-limiting step in the synthesis of monounsaturated fatty acids, such as oleic acid (OA), and is critical for ZIKV infection of Huh7 cells, likely through production of OA^35,42^. In contrast, treatment of moDC with DMHCA and either OA or cholesterol added 1 h after ZIKV infection revealed that exogenous cholesterol, but not OA, fully reversed the inhibitory effect of DMHCA on ZIKV infection (Fig. 5f and Supplementary Fig. 6g). Thus, DMHCA inhibition of ZIKV infection is mediated, at least in part, via a reduction in cellular cholesterol levels. To determine whether this might involve inhibition of SREBP-mediated activation of cholesterol synthesis genes and/or LXR-mediated stimulation of cholesterol efflux, we treated moDCs with GW3965, a non-sterol agonist of LXR TFs, which induce genes involved in cholesterol efflux. Treatment of moDCs with GW3965 had no significant effect on ZIKV infection (Supplementary Fig. 6h), suggesting that DMHCA most likely inhibits infection by suppressing SREBP-dependent cholesterol synthesis. To test this, we silenced *SREBF1* and *SREBF2*, the genes encoding SREBP1 and SREBP2, in moDCs by transfection with gene-specific siRNAs and validated knockdown by qRT-PCR of *SREBF1 and SREBF2* mRNA levels and western blot analysis of SREBP1 and SREBP2 protein levels (Supplementary Fig. 7a–d). Knockdown of *SREBF2* reduced the production of infectious viral particles to ~50% of control levels, whereas *SREBF1* knockdown had no effect (Fig. 5g). Although SREBP2 knockdown can activate type I IFN signaling and ISG transcription in HMDMs^43^, qRT-PCR analysis of moDCs revealed that *SREBF1* or *SREBF2* knockdown had no effect on expression of the ISG *IFIT1* in ZIKV+ or ZIKV− moDCs (Supplementary Fig. 7a, b). Thus, these results demonstrate a crucial role for SREBP2-dependent cholesterol production in the mechanism of ZIKV infection of primary human moDCs.

## Discussion

ZIKV represents an important pathogen that not only generates severe sequelae of infection, but also serves as a platform to understand the pathophysiology of neurotropic viruses. In this study, we interrogated the mechanistic underpinnings of ZIKV infection of primary human moDCs by leveraging a novel method of genomic profiling that detects changes in host gene expression specifically in infected but not uninfected neighboring cells. Only 176 genes were specifically upregulated by ZIKV infection, and among these, lipid metabolism was the most enriched transcriptional pathway. ZIKV promoted expression of lipid metabolism genes by increasing SREBP TF binding to and transcriptional initiation of these genes. Pharmacologic or genetic inhibition of SREBP2 decreased ZIKV infection of moDCs by limiting cellular cholesterol content, which is likely to suppress ZIKV infection at the replication, assembly, and/or budding stages. Thus, ZIKV promotes activation of SREBP2-dependent cholesterol synthesis to infect human moDCs, thereby revealing a novel ZIKV-specific therapeutic strategy.

Flaviviruses have evolved diverse mechanisms to hijack cellular lipids to enhance viral entry, replication, and egress^44–50^. West Nile virus infection increases cholesterol trafficking from the plasma membrane to viral replication complexes^51,52^. DENV infection induces redistribution of the FAS complex to replication sites to increase local fatty acid production and activates lipophagy to break down lipid droplets^53–55^. In contrast to the effects of ZIKV infection, DENV2-infected moDCs did not exhibit upregulated transcription of cholesterol and fatty acid synthesis enzymes when assessed at peak infection. It is possible that DENV stimulates lipid gene transcription in moDCs at very early time points, similar to *SCD* expression in Huh7 cells^42^. However, our data from Huh7.5 cells suggest that transcriptional responses to ZIKV infection in hepatocytes, which play a central role in lipid metabolism and express lipid biosynthesis genes at high levels, may differ from the responses in innate immune cells. Alternatively, like other flaviviruses, DENV may alter lipid metabolism by post-transcriptional mechanisms, whereas ZIKV activates SREBP TFs to increase de novo lipid synthesis.

Type I IFN and viral infection downregulates the transcriptional activity of SREBP, in part by inhibiting its processing^56–59^. However, we found that ZIKV activates SREBP TFs in infected moDCs. Our finding may be explained by the observation that sterol and fatty acid metabolic networks can modulate the functions of immune cells^58,60–62^. Decreasing flux through the SREBP2 cholesterol biosynthetic pathway can spontaneously activate type I IFN response and augment antiviral immunity^43^. Our results comparing ZIKV infection in moDCs vs NPCs, together with publicly available databases indicating that baseline cholesterol and fatty acid expression is comparatively lower in innate immune cells and tissues relative to non-immune cells, suggests that maintenance of SREBP TF activity at low levels in innate immune cells contributes to IFN and antiviral signaling. By activating SREBP TFs, ZIKV may not only induce the production of lipids required for the viral replication cycle but also increase de novo cholesterol synthesis that can suppress antiviral immune responses.

Pharmacologic agents that can inhibit SREBP activity, such as 25-hydroxycholesterol (25HC), nordihydroguaiaretic acid, PF-429242, AM580, and fatostatin, inhibit the activity of many viruses^47,63–73^. Like 25HC, DMHCA also blocks SREBP2 activation, presumably by limiting SREBP–SCAP transport out of the ER^41^. In contrast with 25HC, which appears to primarily block ZIKV entry by altering lipids in the plasma membrane^69^, the majority of the antiviral activity of DMHCA appears to be post-entry. Thus, DMHCA and 25HC employ distinct mechanisms to block ZIKV infection. ZIKV infection of moDCs was inhibited by knockdown of *SREBF2*, but not *SREBF1*, at least at the knockdown efficiencies achieved here, which suggests that DMHCA blocks ZIKV in part by inhibiting SREBP2 activation. A recent parallel genome-wide CRISPR screen also identified *SREBF2*, but not *SREBF1*, as a key host factor in SARS-CoV-2 and HCoV-OC43 infection of Cas9-expressing Huh7.5 hepatoma cells^74^. Thus, SREBP2, but not SREBP1, appears to play an important role in replication of these viruses. Whether SREBP2 is activated in a direct or indirect manner in ZIKV+ moDCs is under investigation.

Serum from ZIKV-infected individuals contain higher than normal levels of phospholipids, including phosphatidylethanolamine and plasmenyl-phosphatidylethanolamine^75,76^. Quantitative analyses of lipid species during ZIKV infection in multiple human cell types have identified significant alterations in fatty acids and phospholipids^77,78^. In human NPCs, ZIKV increases ceramide levels^78^, while in human placental explants, ZIKV infection increases cellular neutral lipids, including fatty acids and certain phospholipids^77^. In both of these examples, the lipid alterations created a favorable environment for ZIKV infection. In our analysis, ZIKV+ moDCs displayed upregulated transcription of enzymes involved in the synthesis of both cholesterol and fatty acids, which can independently stimulate phospholipid biosynthesis^79,80^. Additionally, ZIKV infection of moDCs upregulated *LPIN1*, which catalyzes the conversion of phosphatidic acid to diacylglycerol, a key precursor for the synthesis of triglyceride, phosphatidylcholine, and phosphatidylethanolamine. Thus, transcriptional upregulation of selected SREBP target genes and phospholipid biosynthetic enzymes could contribute to the proviral lipid changes observed in human NPCs, placental explants, and individuals with acute ZIKV infection.

Our infection-specific genomic profiling approach identified ZIKV-regulated transcriptional programs by comparing responses in pure populations of ZIKV+ vs mock-infected vs uninfected bystander cells. The results of the present study, together with our earlier analysis of ZIKV infection of macrophages^17^, show that accurate identification of cellular responses to viral infection can be obscured when infected and bystander cells are analyzed together. In support of our approach, we observed ZIKV-induced induction of *DHCR7*, *SCD*, and *HMGCR* only in purified ZIKV+ cells, and not in a mixed population. Although RNA-seq can identify the functional importance of gene expression changes during viral infections, it is less useful for determining the TFs responsible for those changes. We demonstrate here for the first time that csRNA-seq can be applied to host–pathogen interactions to identify the key regulatory TFs driving host transcriptional responses. We further show that csRNA-seq can be performed on formaldehyde-fixed and FACS-sorted cells. As such, in contrast to other nascent methods, like global run-on sequencing (GRO-seq)^81^ and precision run-on sequencing (PRO-seq)^82^, csRNA-seq allows fixation that can inactivate infectious starting material as well as FACS separation of complex populations based on cell type or infection status. This method also requires significantly less expertize and starting material while still identifying differentially regulated TSRs at all regulatory elements, including promoters and enhancers, at single-nucleotide resolution, which boosts the power to identify regulatory TFs^19^. These advantages make csRNA-seq a key technique to study transcription initiation and gene regulation in infectious diseases and other conditions involving heterogeneous populations. Integrating transcriptional analysis of pure populations of virally infected cells with csRNA-seq to identify regulatory TFs and follow-up confirmation by ChIP-seq thus represents a powerful approach to studying virally induced gene regulatory networks in primary human cells or tissues.

In summary, we performed unbiased genomic profiling of pure populations of ZIKV-infected moDCs and demonstrated that ZIKV infection upregulates a small set of genes that are highly enriched for lipid biosynthetic enzymes. ZIKV+ cells demonstrated elevated SREBP TF binding at lipid genes that was associated with increased nascent-like RNA synthesis. Pharmacologic antagonism or siRNA-mediated knockdown of SREBP2 inhibited ZIKV infection in moDCs. Thus, targeting host lipid metabolism could provide antiviral therapies for many important human pathogenic viruses that lack FDA-approved therapies. Understanding how distinct viruses manipulate host lipids will be critical for developing optimal lipid-targeting therapies.

## Methods

### Reagents

Key reagents, antibodies, primers, and probes used in this study are listed in Supplementary Table S1.

### Cell culture

BHK-21 cells (ATCC, #CCL 10) were grown in MEMα medium supplemented with 10% fetal bovine serum (FBS), 1% penicillin/streptomycin, and 1% HEPES. C6/36 *Aedes albopictus* mosquito cells (ATCC, # CRL-1660) were cultivated in Leibovitz’s L-15 supplemented with penicillin, streptomycin, HEPES, and 10% fetal bovine serum (Gemini Bio-Products). C6/36 infection was performed in 5% fetal bovine serum. Cells were grown at 28 °C without CO_2_^83,84^. Huh7.5 cells were obtained from APATH, LLC and cultured in DMEM (Invitrogen) supplemented with 10% FBS, and 1% penicillin/streptomycin. The human-induced pluripotent cell line WT-126 was derived in the lab of Alysson Muotri^85,86^. WT-126 cells were incubated in Minimum Essential Medium (Invitrogen) supplemented with 10% fetal bovine serum (HyClone Laboratories). To obtain NPCs, embryoid bodies (EBs) were formed by mechanical dissociation of cell clusters and plating onto low-adherence dishes in hESC medium without FGF2 for 5-7 days. After that, EBs were plated onto poly-ornithine/laminin (Sigma)-coated dishes in DMEM/F12 (Invitrogen) plus N2. Rosettes were obtained after 7 days and dissociated with Accutase and cultured on Geltrex-coated plates in neurobasal medium supplemented with 1× B27, 1× N2, 1× GlutaMAX, pyruvate, penicillin/streptomycin, and 20 ng/ml each EGF and FGF. Homogeneous populations of NPCs were achieved after 1–2 passages with Accutase in the same condition. NPCs were passaged by dissociation with Accutase. All mammalian cells were cultured in a 5% CO_2_ humidified atmosphere.

### Differentiation of primary human monocytes to moDCs

All uses of human material have been approved by the La Jolla Institute Institutional Review Board (IRB), Protocol VD-057-0217, and the UCSD IRB, Protocol 181624. All recruited volunteers provided written informed consent. Human blood was obtained from healthy volunteers, deidentified, centrifuged over Histopaque without acceleration, and brake at 300×*g* for 30 min at 4 °C, the buffy coat was removed and washed once, and red blood cells were lysed with molecular grade water. Monocytes were purified by negative selection using the human Pan Monocyte Isolation Kit (Miltenyi Biotec) according to the manufacturer’s recommendations. To generate moDCs, monocytes were seeded in six-well polystyrene plates at 1.5 × 10^6^ cells/ml in complete moDC medium (RPMI 1640 supplemented with GlutaMAX, 1% penicillin/streptomycin, 2.5% HEPES buffer, 100 ng/ml recombinant human granulocyte-macrophage colony-stimulating factor, and 100 ng/ml recombinant human interleukin 4 and incubated for 7 days at 37 °C. The medium was changed every 2–3 days.

### Viruses

ZIKV PRVABC59, an Asian lineage strain isolated in 2015 from an individual in Puerto Rico^87^, and DENV2 UIS353, a clinical isolate collected in 2004 in Bucaramanga Santander, Colombia from acute sera from an infected patient, were obtained from the World Reference Center for Emerging Viruses and Arboviruses. ZIKV-EGFP-BeH819015^23^ was provided by Dr. Tariq Rana (UCSD). ZIKV BeH819015 was isolated from an individual in Brazil in 2015^88^. ZIKV SD001, an Asian lineage strain isolated by our laboratory in San Diego in 2016 from an acutely infected individual returning from Venezuela^17^. ZIKV strain FSS13025, an Asian lineage strain isolated in 2010 from a pediatric patient^89^, was obtained from the World Reference Center for Emerging Viruses and Arboviruses. All viruses were cultured using C6/36 *Aedes albopictus* mosquito cells as described previously^83,84^ and were titrated using a BHK-21 cell-based FFA as described previously^90^.

### ZIKV/DENV infections

On day 7 of differentiation, moDCs were infected with ZIKV SD001, ZIKV PRVABC59, ZIKV-EGFP-BeH819015, or DENV UIS353 at MOIs indicated in the text. moDCs were mixed with virus, incubated for 2 h at 37 °C, washed with phosphate-buffered saline (PBS), and then resuspended in fresh medium and incubated for the indicated times at 37 °C. NPCs were infected as in^91^. Briefly, cells were plated one day before infection and infected at a MOI of 1 for 2 h, then washed with PBS and incubated for 24 h in supplemented Neurobasal medium described above. Huh7.5 cells were seeded one day prior to infection, infected at an MOI of 1 with ZIKV PRVABC59 for 2 h, washed with PBS, and supplemented with Huh7.5 medium described above for 22 additional hours.

### Separation of virally infected and uninfected cells by cell sorting for RNA-seq, csRNA-seq, and qRT-PCR

moDCs were infected with ZIKV or DENV2 for 24 h at MOIs of 0.5 and 1, respectively. Huh7.5 cells and NPCs were infected with ZIKV for 24 h at an MOI of 1. The cells were then collected, incubated with Zombie Violet™ Fixable Viability stain (BioLegend) for 20 min at 4 °C, washed, fixed with 4% paraformaldehyde, and permeabilized with 0.1% saponin in the presence of RNasin ribonuclease inhibitor (400 U/ml). The cells were centrifuged, resuspended in wash buffer (PBS containing 0.2% bovine serum albumin [BSA], 0.1% saponin, and 400 U/ml RNasin), and incubated for 5 min with human Fc Blocker in staining buffer (1% BSA, 0.1% saponin, 1600 U/ml RNasin) at 4 °C. The cells were then incubated with Alexa Fluor 647-conjugated 4G2 (anti-flavivirus group antigen), incubated for 30 min at 4 °C, and centrifuged at 1000×*g* for 3 min at 4 °C. The cells were washed in wash buffer, re-centrifuged, and finally resuspended in sort buffer (PBS containing 0.5% BSA and 1600 U/ml of RNasin). ZIKV+, ZIKV−, DENV+, and DENV− cells were sorted using a FACSAria (BD Biosciences) or MA900 (Sony) sorter.

### RNA-seq and csRNA-seq

Sorted cells were centrifuged at 4 °C, and RNA was isolated from the cell pellet RecoverAll Total Nucleic Acid Isolation Kit (Ambion, AM1975). Starting at the protease digestion step, all steps were performed according to the manufacturer’s recommendations with the following exceptions. Cells were incubated in digestion buffer supplemented with RNasin Plus for 3 h at 50 °C. After in-column DNase treatment, RNA was eluted and the quality was determined using BioAnalyzer Eukaryote Total RNA Pico Chip. RNA libraries were generated using the TruSeq Stranded Total RNA-seq Kit (Illumina) according to the manufacturer’s instructions, and then single-end sequenced for 51 cycles on an Illumina HiSeq 2000 or NextSeq 500 according to the manufacturer instructions. csRNA-seq was performed as previously described^19^. Briefly, small RNAs of ~20–60 nucleotides were size selected with generous spacing from >1 µg of total RNA with generous spacing on a 15% acrylamide, 7 M urea, and 1× TBE gel (Invitrogen EC6885BOX), eluted, and precipitated overnight at −20 °C. Because RNA fragmentation occurs with de-crosslinking, input libraries were generated to eliminate potential false positives and ensure accurate TSS peak calling. csRNA-seq libraries were twice cap selected prior to decapping and libraries were generated as described above. Input libraries were treated with the RppH pyrophosphatase (NEB M0356) prior to adapter ligation to include the whole repertoire of small RNAs with 3′-OH. Samples were quantified by Qbit (Invitrogen) and sequenced using the Illumina NextSeq 500 platform using 75 cycles of single-end sequencing according to the manufacturer’s instructions.

### Separation of ZIKV+ and ZIKV− cells by cell sorting for ChIP-seq

moDCs were crosslinked by incubation at room temperature with 4 mM disuccinimidyl glutarate in PBS for 30 min followed by 1% formaldehyde for 15 min, and then quenched with 0.125 M glycine. moDCs were prepared for cell sorting as described above except that RNasin was replaced with 1× cOmplete protease inhibitors (Roche) in all buffers. After sorting, the cell populations were washed, divided into 5 × 10^5^ cell aliquots, centrifuged, snap-frozen, and stored at −80 °C.

### SREBP ChIP-seq

Samples of 5 × 10^5^ cells were resuspended on ice in 130 µl RLNR1 lysis buffer (20 mM Tris/HCl pH 7.5, 150 mM NaCl, 1 mM EDTA, 0.5 mM EGTA, 0.1% SDS, 0.4% sodium deoxycholate, 1% NP-40 Alternative, 0.5 mM DTT, and 1× protease inhibitor cocktail), and transferred to microtubes with an AFA Fiber (Covaris, MA). All subsequent steps were performed at 4 °C. Chromatin was sheared by sonication in a 96 Place microTUBE Rack (Covaris #500282) using a Covaris E220 focused-ultrasonicator (Covaris) for 20 cycles with the following settings: time, 60 s; duty, 5; PIP, 140; cycles, 200; amplitude, 0.0; velocity, 0.0; dwell, 0.0. Samples were recovered and spun at maximum speed for 10 min and the pellet was discarded. An aliquot of 1% of the sample volume was reserved as DNA input control and stored at −20 °C, and the remaining supernatant was transferred to PCR strips and brought up to a volume of 200 µl using RLNR1 lysis buffer. To prepare Ab-coupled beads for ChIP, protein A/G Dynabeads (20 μl per sample) were washed twice with 1 ml 0.5% BSA in TET/0.1% (10 mM Tris-HCl, pH 8, 1 mM EDTA, 0.1% Tween 20), and resuspended in the same buffer. Anti-SREBP Abs (Santa Cruz Biotechnology sc-8984X, Thermo Fisher Scientific PA1-337, R&D Systems AF7119) were added to the beads and rotated for 1 h at RT. The supernatant was then removed, and the Dynabeads were collected using a magnet, washed once with 0.1% BSA in TET/0.1%, and resuspended in 10 µl RIPA buffer per sample. For ChIP, 10 μl of prepared Ab-Dynabeads was added to each sample and rotated overnight at 4 °C. The beads were then washed 3 times with Wash Buffer 1 (20 mM Tris-HCl, pH 7.4, 150 mM NaCl, 2 mM EDTA, 0.1% SDS, 1% Triton X-100), three times with Wash Buffer 3 (10 mM Tris-HCl, pH 7.4 250 mM LiCl, 1 mM EDTA, 1% Triton X-100, 0.7% sodium deoxycholate), 3 times with TET/0.2% (as above except 0.2% Tween 20), and once with TE-NaCl (10 mM Tris-HCl, pH 8, 1 mM EDTA, 50 mM NaCl). Finally, Dynabeads were resuspended in 25 μl TT (10 mM Tris-HCl, pH 8, 0.05% Tween 20). Input samples were resuspended in 25 μl TT and libraries were generated in parallel with ChIP samples. Library NEBNext End Prep and Adaptor Ligation were performed using NEBNext Ultra II DNA Library Prep kit (New England BioLabs) according to the manufacturer’s instructions with barcoded adapters (NextFlex, Bioo Scientific). Libraries were incubated with RNase A and proteinase K at 55 °C for 1 h and then at 65 °C overnight. Libraries were PCR amplified for 14 cycles with NEBNext High Fidelity 2X PCR MasterMix (New England BioLabs, NEBM0541). Libraries were size selected for 225–350 bp fragments by gel extraction (10% TBE gels, Life Technologies) and were single-end sequenced for 51 cycles on an Illumina HiSeq 4000 (Illumina, San Diego, CA).

### Drug treatments

moDCs were incubated with the indicated concentrations of *N*,*N*-dimethyl-3β-hydroxycholenamide or ethanol vehicle for 4 h before or 2.5 h after ZIKV infection. GW3965 at 1 μM was added to moDCs at 2.5 h after infection. Cholesterol–methyl-β-cyclodextrin at 25 μg/ml or OA–BSA at 75 μM or 300 μM were added at 1 h post-infection together with DMHCA. After addition, the compounds were present throughout the 24 h infection period.

### qRT-PCR of unspliced and spliced *XBP1*

After 24 h infection, moDCs were sorted and total RNA was isolated as described above. As a positive control, moDCs were incubated with tunicamycin (2 μg/ml) for 5 h to induce ER stress. Total RNA was isolated from the moDC populations using the Zymo Quick RNA isolation kit with in-column DNase digestion according to the manufacturer’s instructions. RNA was reverse transcribed using a Bio-Rad iScript cDNA synthesis kit. Quantitative PCR was performed with iTaq Universal SYBR Green Supermix (Bio-Rad) and analyzed on an Applied Biosystems 7300 Real-Time PCR system (Invitrogen). qPCR primers for unspliced (u) *XBP1*, spliced (s) *XBP1*, and total (t) *XBP1* (common region of s/uXBP1) can be found in supplementary table 4^92^.

### Western blot analysis

Equal numbers of moDCs cells were collected for each condition and were lysed using RIPA buffer with the presence of cOmplete protease inhibitors (Roche). Samples were then heated to 70 °C in LDS sample buffer (Invitrogen) for 10 min. Protein lysates were separated by Bolt 4-12% Bis-Tris plus gel, electrophoretically transferred to a PVDF membrane, and immune-blotted at 4 °C overnight with antibodies (1:1000) against SREBP1, SREBP2, GAPDH. Membranes were then incubated with an HRP-conjugated second antibody (Rabbit or Goat, 1:10,000) for 1 h at room temperature followed by detection by chemiluminescence (Bio-Rad). Images were collected by Biorad Chemidoc MP imaging system and analyzed with Image Lab 6.0.1.

### MTS cell proliferation/cytotoxicity assay

Approximately 100,000 moDCs were plated in a sterile 96-well round-bottom culture in 50 μl moDC medium. Then 50 μl of moDC media containing 2× concentration of lipid inhibitor or vehicle was added and gently mixed. Cells were incubated at 37 °C. After 28 h, 20 μl of reagent containing 3-(4,5-dimethylthiazol-2-yl)−5-(3-carboxymethoxyphenyl)−2-(4-sulfophenyl)−2H-tetrazolium (MTS) (Promega, CellTiter 96 Aqueous One Solution Cell Proliferation Assay System G3582) was added, incubated for 1 to 4 h at 37 °C, and absorbance measured (490 nm) using a plate reader (uQuant, Bio-Tek Instruments, Inc.).

### siRNA-mediated gene silencing

Transfection mixes of SMARTpool ON-TARGETplus *SREBF1*-targeting*, SREBF2*-targeting, or Non-targeting Pool siRNAs were prepared using the StemFect RNA Transfection kit (Reprocell) according to the manufacturer’s instructions and incubated for 20 min at room temperature. moDCs were then reverse transfected as previously described^93^. Briefly, aliquots of transfection mix (150 pmol siRNA/well) were placed on one side of the well in six-well plates and 1 ml of moDCs (1.5 × 10^6^/ml) was added directly onto the transfection mix and incubated at 37 °C for 4 h. Transfection was stopped by addition of 2 ml of complete medium (RPMI 1640 supplemented with GlutaMAX, 1% penicillin/streptomycin, 2.5% HEPES buffer, 100 ng/ml recombinant human granulocyte-macrophage colony-stimulating factor, and 100 ng/ml recombinant human interleukin 4) and the cells were incubated for an additional 24 h before viral infection.

### Flow cytometry of infected moDCs and neutral lipids in moDCs

Following viral infection, moDCs were collected, stained with Zombie Violet™ Fixable Viability stain (BioLegend), washed, fixed, and permeabilized using BD Cytofix/Cytoperm reagents, and then intracellularly stained with FITC- or AF647-conjugated 4G2 mAb. Cells were washed twice with BD Perm/Wash Buffer and resuspended in FACS buffer. To quantify neutral lipid content, moDCs were collected, stained with BODIPY 493/503, and then treated as described above. The percentage uninfected and infected cells and the neutral lipid content in uninfected and infected cells were quantified by flow cytometry using a LSRII flow cytometer (BD Biosciences) or MA900 cell sorter (Sony) and Flowjo 10.8 software.

### Quantification of intracellular and extracellular virus production by FFA

To quantify intracellular particles, moDCs were collected, suspended in PBS, lysed by 4 cycles of freeze-thaw in dry ice and a 37 °C water bath, and centrifuged. The supernatants were collected and subjected to FFA. To quantify extracellular particles, the culture supernatants were collected, clarified by centrifugation, and subjected to FFA directly. FFA was performed using BHK-21 cells as previously described^90^. Briefly, BHK-21 cells were plated (2.0 × 10^5^ cells/well) in 24-well plates and incubated overnight at 37 °C. Undiluted or 10-fold serially diluted samples were added to the cells and the plates were incubated for 1 h at 37 °C. The supernatant was then removed, and the cell monolayers were overlaid with CMC-medium and incubated at 37 °C for 3 days. The cells were then fixed, permeabilized, incubated with 4G2 mAb, and incubated with horseradish peroxidase-conjugated goat anti-mouse IgG secondary Ab. Finally, True-Blue peroxidase substrate was added to the cells and foci were counted. Virus levels were expressed as focus-forming units per ml (FFU/ml).

### qRT-PCR for quantification of human and viral RNA

Total RNA was isolated from unfixed moDCs using the Quick RNA isolation kit (Zymo Research) with in-column DNase digestion according to the manufacturer’s instructions. RNA from fixed FACS-isolated moDCs was prepared as described above for RNA-seq experiments. RNA was reverse transcribed using iScript cDNA Synthesis kit (Bio-Rad), and qPCR was performed with iTaq Universal SYBR Green Supermix (Bio-Rad) on an Applied Biosystems 7300 Real-Time PCR system (Invitrogen). Specific primers are listed in Table S1.

### NGS data preprocessing

FASTQ files from sequencing experiments were mapped to the UCSC genome build GRCh38/hg38 (for human) and access KU955593.1 (for the ZIKV genome). FASTQ files for csRNA-seq experiments were first trimmed to remove the 3′ sequencing adapter using homerTools (*homerTools trim −3 AGATCGGAAGAGCACACGTCT -mis 2 -minMatchLength 4 -min 20*). STAR with default parameters was used to map RNA-seq and csRNA-seq data. Bowtie2 with default parameters was used to map ChIP-seq data. HOMER was used to convert uniquely aligned reads into “tag directories” for further analysis.

### Integrated NGS data analysis

RNA-seq or csRNA-seq reads aligned to a combined GRCh38/hg38 and ZIKV genome (KU955593.1) were used to calculate the percentage of reads aligned to the ZIKV genome: ([# reads aligned to ZIKV genome / # aligned reads to hg38+ZIKV genomes] × 100 − average numbers of reads aligning to ZIKV genome in mock-infected cells). RNA-seq reads aligned to the GRCh38/hg38 assembly were used to generate gene expression fragments per kilobase of exon per million mapped fragments (FPKM) values using HOMER^94^. To measure gene expression, HOMER’s analyzeRepeats.pl utility was used to quantify reads in transcript exons defined by GENCODE. Differentially expressed genes and regularized logarithm (rlog) normalization values for each gene were calculated using DESeq2 while accounting for individual donors in the design matrix. csRNA-seq reads aligned to the GRCh38/hg38 assembly from three replicates per condition (mock, ZIKV−, ZIKV+) were combined and TSRs identified using HOMER *findcsRNATSS.pl* with the corresponding combined input and RNA-seq tag directories. SREBP ChIP-seq peaks were called using tags from three replicates per condition with input DNA as background using HOMER’s *getDifferentialPeaksReplicates.pl* using the “-style factor” (fivefold enrichment over background and FDR < 0.001). Differentially regulated TSRs/peaks between conditions were calculated by first merging features from each condition (or assay) into the union of nonredundant features using *mergePeaks*. Then raw read counts associated with each feature across all experiments was quantified with *annotatePeaks.pl* and significantly differentially enriched TSRs/peaks determined by DESeq2 accounting for donor-matched samples in the design matrix (csRNA-seq: >2-fold, <0.01 FDR, ChIP-seq >1.5-fold, <0.1 FDR)^95^. Known motif enrichment and de novo motif discovery were performed using HOMER’s *findMotifsGenome.pl* using default parameters. When analyzing csRNA-seq TSRs, motifs were searched from −200 to +50 relative to the primary TSS of a TSR (i.e., site with the highest csRNA-seq read count). SREBP peaks were analyzed from −50 to +50 relative to the center of the peaks, reflecting the locations where TFs and collaborating TF motifs are located. Normalized histograms, heatmaps, and read count totals at TSS clusters or ChIP-seq peaks were calculated using HOMER’s *annotatePeaks.pl* and reported relative to a total of 10^7^ uniquely aligned reads per experiment. Functional enrichment calculations were performed on differentially expressed genes (RNA-seq), promoter proximal (±500 bp from TSS) csRNA-seq TSRs or SREBP ChIP-seq peaks using Metascape^96^. Fold-change values were clustered using Cluster 3.0^97^ and visualized using Java TreeView^98^. For comparison of gene expression in different cell types under mock and ZIKV+ conditions, strand-specific FPKM were calculated using HOMER’s *analyzeRepeats.pl* with the following options -condenseGenes -count exons, and log2 transformed after adding a pseudocount of 1 to reduce the variance associated with fluctuations in low expression values and to avoid taking the log of zero.

### Gene expression in human tissues and cell types

Expression of lipid metabolism genes in human tissues and cell types was determined using Human Protein Atlas version 21.0 (proteinatlas.org) and GTEx Analysis Release version 8 (gtexportal.org)^22,99^.

### Statistical Analysis

All data were analyzed and graphs were plotted using Excel or Prism 8.4.3 (GraphPad Software). Data were compared using one-way analysis of variance with indicated correction for multiple comparisons or Student’s *t* test, as stated in the legends. Data are presented as the mean ± standard deviation (SD) of cells isolated from at least three individuals and/or at least three experiments. A *P* value <0.05 was considered significant.

### Reporting summary

Further information on research design is available in the Nature Research Reporting Summary linked to this article.

## Supplementary information

Supplementary Information

Reporting Summary

## Data Availability

The RNA-seq, csRNA-seq, and ChIP-seq data described in this manuscript have been deposited at National Center for Biotechnology Information Gene Expression Omnibus (GEO) under the accession codes GSE161783 and GSE118305. The processed qRT-PCR, flow cytometry, western blot, and FFU data generated in this study are provided in the Source Data file. Source data are provided with this paper.

## Source data

Source Data

## References

[1] Brasil P (2016). Guillain-Barre syndrome associated with Zika virus infection. Lancet.

[2] Bautista LE, Sethi AK (2016). Association between Guillain-Barre syndrome and Zika virus infection. Lancet.

[3] Mlakar J (2016). Zika virus associated with microcephaly. N. Engl. J. Med..

[4] Rasmussen SA, Jamieson DJ, Honein MA, Petersen LR (2016). Zika virus and birth defects–reviewing the evidence for causality. N. Engl. J. Med..

[5] de Araujo TVB (2018). Association between microcephaly, Zika virus infection, and other risk factors in Brazil: final report of a case-control study. Lancet Infect. Dis..

[6] Martines RB (2016). Pathology of congenital Zika syndrome in Brazil: a case series. Lancet.

[7] Lucey D, Cummins H, Sholts S (2017). Congenital Zika Syndrome in 2017. JAMA.

[8] Meneses JDA (2017). Lessons learned at the epicenter of Brazil’s congenital zika epidemic: evidence from 87 confirmed cases. Clin. Infect. Dis..

[9] Sun X (2017). Transcriptional changes during naturally acquired Zika virus infection render dendritic cells highly conducive to viral replication. Cell Rep..

[10] Hou W (2012). Viral infection triggers rapid differentiation of human blood monocytes into dendritic cells. Blood.

[11] Malissen B, Tamoutounour S, Henri S (2014). The origins and functions of dendritic cells and macrophages in the skin. Nat. Rev. Immunol..

[12] Bowen JR (2017). Zika virus antagonizes type I interferon responses during infection of human dendritic cells. PLoS Pathog..

[13] Singh PK (2018). Determination of system level alterations in host transcriptome due to Zika virus (ZIKV) infection in retinal pigment epithelium. Sci. Rep..

[14] Tiwari SK (2017). Zika virus infection reprograms global transcription of host cells to allow sustained infection. Emerg. Microbes Infect..

[15] Park T, Kang MG, Baek SH, Lee CH, Park D (2020). Zika virus infection differentially affects genome-wide transcription in neuronal cells and myeloid dendritic cells. PLoS One.

[16] Lima MC (2019). The transcriptional and protein profile from human infected neuroprogenitor cells is strongly correlated to Zika virus microcephaly cytokines phenotype evidencing a persistent inflammation in the CNS. Front. Immunol..

[17] Carlin AF (2018). Deconvolution of pro- and antiviral genomic responses in Zika virus-infected and bystander macrophages. Proc. Natl Acad. Sci. USA.

[18] Carlin AF, Shresta S (2019). Genome-wide approaches to unravelling host-virus interactions in Dengue and Zika infections. Curr. Opin. Virol..

[19] Duttke SH, Chang MW, Heinz S, Benner C (2019). Identification and dynamic quantification of regulatory elements using total RNA. Genome Res..

[20] Grant A (2016). Zika virus targets human STAT2 to inhibit Type I interferon signaling. Cell Host Microbe.

[21] Miner JJ, Diamond MS (2017). Zika virus pathogenesis and tissue tropism. Cell Host Microbe.

[22] Uhlen M (2015). Proteomics. Tissue-based map of the human proteome. Science.

[23] Mutso M (2017). Reverse genetic system, genetically stable reporter viruses and packaged subgenomic replicon based on a Brazilian Zika virus isolate. J. Gen. Virol..

[24] Quicke KM (2016). Zika virus infects human placental macrophages. Cell Host Microbe.

[25] Lam MT (2013). Rev-Erbs repress macrophage gene expression by inhibiting enhancer-directed transcription. Nature.

[26] Eberle D, Hegarty B, Bossard P, Ferre P, Foufelle F (2004). SREBP transcription factors: master regulators of lipid homeostasis. Biochimie.

[27] Amemiya-Kudo M (2002). Transcriptional activities of nuclear SREBP-1a, -1c, and -2 to different target promoters of lipogenic and cholesterogenic genes. J. Lipid. Res..

[28] Osborne TF, Espenshade PJ (2009). Evolutionary conservation and adaptation in the mechanism that regulates SREBP action: what a long, strange tRIP it’s been. Genes Dev..

[29] Jackson SM, Ericsson J, Mantovani R, Edwards PA (1998). Synergistic activation of transcription by nuclear factor Y and sterol regulatory element binding protein. J. Lipid Res..

[30] Dooley KA, Millinder S, Osborne TF (1998). Sterol regulation of 3-hydroxy-3-methylglutaryl-coenzyme A synthase gene through a direct interaction between sterol regulatory element binding protein and the trimeric CCAAT-binding factor/nuclear factor Y. J. Biol. Chem..

[31] Reed BD, Charos AE, Szekely AM, Weissman SM, Snyder M (2008). Genome-wide occupancy of SREBP1 and its partners NFY and SP1 reveals novel functional roles and combinatorial regulation of distinct classes of genes. PLoS Genet..

[32] Park, S. M., Kang, T. I. & So, J. S. Roles of XBP1s in transcriptional regulation of target genes. *Biomedicines* **9**, 10.3390/biomedicines9070791 (2021). 10.3390/biomedicines9070791 PMC8301375 34356855

[33] Read, A. & Schroder, M. The unfolded protein response: an overview. *Biology (Basel)* **10**, 10.3390/biology10050384 (2021). 10.3390/biology10050384 PMC8146082 33946669

[34] Kolpikova, E. P. et al. IRE1alpha promotes zika virus infection via XBP1. *Viruses* **12**, 10.3390/v12030278 (2020). 10.3390/v12030278 PMC7150863 32138181

[35] Huang, Y. et al. Inositol-requiring enzyme 1alpha promotes Zika virus infection through regulation of stearoyl coenzyme a desaturase 1-mediated lipid metabolism. *J. Virol.* **94**, 10.1128/JVI.01229-20 (2020). 10.1128/JVI.01229-20 PMC7654271 32967957

[36] Ye, J. & DeBose-Boyd, R. A. Regulation of cholesterol and fatty acid synthesis. *Cold Spring Harb. Perspect. Biol.* **3**, 10.1101/cshperspect.a004754 (2011). 10.1101/cshperspect.a004754 PMC3119913 21504873

[37] Brown MS, Radhakrishnan A, Goldstein JL (2018). Retrospective on cholesterol homeostasis: the central role of scap. Annu. Rev. Biochem..

[38] Hannah VC, Ou J, Luong A, Goldstein JL, Brown MS (2001). Unsaturated fatty acids down-regulate srebp isoforms 1a and 1c by two mechanisms in HEK-293 cells. J. Biol. Chem..

[39] Horton JD, Goldstein JL, Brown MS (2002). SREBPs: activators of the complete program of cholesterol and fatty acid synthesis in the liver. J. Clin. Invest.

[40] Quinet EM (2004). Gene-selective modulation by a synthetic oxysterol ligand of the liver X receptor. J. Lipid Res..

[41] Muse ED (2018). Cell-specific discrimination of desmosterol and desmosterol mimetics confers selective regulation of LXR and SREBP in macrophages. Proc. Natl. Acad. Sci. USA.

[42] Gullberg RC (2018). Stearoly-CoA desaturase 1 differentiates early and advanced dengue virus infections and determines virus particle infectivity. PLoS Pathog..

[43] York AG (2015). Limiting cholesterol biosynthetic flux spontaneously engages type I IFN signaling. Cell.

[44] Pombo JP, Sanyal S (2018). Perturbation of intracellular cholesterol and fatty acid homeostasis during flavivirus infections. Front. Immunol..

[45] Osuna-Ramos JF, Reyes-Ruiz JM, Del Angel RM (2018). The role of host cholesterol during flavivirus infection. Front. Cell Infect. Microbiol..

[46] Abu-Farha, M. et al. The role of lipid metabolism in COVID-19 virus infection and as a drug target. *Int. J. Mol. Sci.* **21**, 10.3390/ijms21103544 (2020). 10.3390/ijms21103544 PMC7278986 32429572

[47] Yuan S (2019). SREBP-dependent lipidomic reprogramming as a broad-spectrum antiviral target. Nat. Commun..

[48] Martin-Acebes MA, Vazquez-Calvo A, Saiz JC (2016). Lipids and flaviviruses, present and future perspectives for the control of dengue, Zika, and West Nile viruses. Prog. Lipid Res..

[49] Leier HC, Messer WB, Tafesse FG (2018). Lipids and pathogenic flaviviruses: an intimate union. PLoS Pathog..

[50] Yan, B. et al. Characterization of the lipidomic profile of human coronavirus-infected cells: implications for lipid metabolism remodeling upon coronavirus replication. *Viruses* **11**, 10.3390/v11010073 (2019). 10.3390/v11010073 PMC6357182 30654597

[51] Samsa MM (2009). Dengue virus capsid protein usurps lipid droplets for viral particle formation. PLoS Pathog..

[52] Mackenzie JM, Khromykh AA, Parton RG (2007). Cholesterol manipulation by West Nile virus perturbs the cellular immune response. Cell Host Microbe.

[53] Heaton NS (2010). Dengue virus nonstructural protein 3 redistributes fatty acid synthase to sites of viral replication and increases cellular fatty acid synthesis. Proc. Natl. Acad. Sci. USA.

[54] Tang WC, Lin RJ, Liao CL, Lin YL (2014). Rab18 facilitates dengue virus infection by targeting fatty acid synthase to sites of viral replication. J. Virol..

[55] Martin-Acebes MA, Blazquez AB, Jimenez de Oya N, Escribano-Romero E, Saiz JC (2011). West Nile virus replication requires fatty acid synthesis but is independent on phosphatidylinositol-4-phosphate lipids. PLoS One.

[56] Blanc M (2011). Host defense against viral infection involves interferon mediated down-regulation of sterol biosynthesis. PLoS Biol..

[57] Reboldi A (2014). Inflammation. 25-Hydroxycholesterol suppresses interleukin-1-driven inflammation downstream of type I interferon. Science.

[58] Robertson KA, Ghazal P (2016). Interferon control of the sterol metabolic network: bidirectional molecular circuitry-mediating host protection. Front. Immunol..

[59] Adams CM (2004). Cholesterol and 25-hydroxycholesterol inhibit activation of SREBPs by different mechanisms, both involving SCAP and Insigs. J. Biol. Chem..

[60] Everts B, Pearce EJ (2014). Metabolic control of dendritic cell activation and function: recent advances and clinical implications. Front. Immunol..

[61] Kelly B, O’Neill LA (2015). Metabolic reprogramming in macrophages and dendritic cells in innate immunity. Cell Res..

[62] Phan AT, Goldrath AW, Glass CK (2017). Metabolic and epigenetic coordination of T cell and macrophage immunity. Immunity.

[63] Waris G, Felmlee DJ, Negro F, Siddiqui A (2007). Hepatitis C virus induces proteolytic cleavage of sterol regulatory element binding proteins and stimulates their phosphorylation via oxidative stress. J. Virol..

[64] Petersen J (2014). The major cellular sterol regulatory pathway is required for Andes virus infection. PLoS Pathog..

[65] Merino-Ramos, T., Jimenez de Oya, N., Saiz, J. C. & Martin-Acebes, M. A. Antiviral activity of nordihydroguaiaretic acid and its derivative tetra-O-methyl nordihydroguaiaretic acid against west Nile Virus and Zika virus. *Antimicrob. Agents Chemother.* **61**, 10.1128/AAC.00376-17 (2017). 10.1128/AAC.00376-17 PMC5527643 28507114

[66] Soto-Acosta R, Bautista-Carbajal P, Syed GH, Siddiqui A, Del Angel RM (2014). Nordihydroguaiaretic acid (NDGA) inhibits replication and viral morphogenesis of dengue virus. Antivir. Res..

[67] Liu SY (2013). Interferon-inducible cholesterol-25-hydroxylase broadly inhibits viral entry by production of 25-hydroxycholesterol. Immunity.

[68] Blanc M (2013). The transcription factor STAT-1 couples macrophage synthesis of 25-hydroxycholesterol to the interferon antiviral response. Immunity.

[69] Li C (2017). 25-hydroxycholesterol protects host against Zika virus infection and its associated microcephaly in a mouse model. Immunity.

[70] Wang, S. et al. Cholesterol 25-hydroxylase inhibits SARS-CoV-2 and other coronaviruses by depleting membrane cholesterol. *EMBO J.* e106057, 10.15252/embj.2020106057 (2020). 10.15252/embj.2020106057 PMC7537045 32944968

[71] Zu, S. et al. 25-Hydroxycholesterol is a potent SARS-CoV-2 inhibitor. *Cell Res.*, 10.1038/s41422-020-00398-1 (2020). 10.1038/s41422-020-00398-1 PMC7431750 32811977

[72] Shibata N (2013). 25-Hydroxycholesterol activates the integrated stress response to reprogram transcription and translation in macrophages. J. Biol. Chem..

[73] Zang R (2020). Cholesterol 25-hydroxylase suppresses SARS-CoV-2 replication by blocking membrane fusion. Proc. Natl Acad. Sci. USA.

[74] Schneider WM (2021). Genome-scale identification of SARS-CoV-2 and pan-coronavirus host factor networks. Cell.

[75] Queiroz A (2019). Lipidomic analysis reveals serum alteration of plasmalogens in patients infected with ZIKA virus. Front. Microbiol..

[76] Melo C (2017). Serum metabolic alterations upon Zika infection. Front. Microbiol..

[77] Chen Q (2020). Metabolic reprogramming by Zika virus provokes inflammation in human placenta. Nat. Commun..

[78] Leier HC (2020). A global lipid map defines a network essential for Zika virus replication. Nat. Commun..

[79] Tabas I (2002). Consequences of cellular cholesterol accumulation: basic concepts and physiological implications. J. Clin. Invest..

[80] Sundler R, Akesson B (1975). Regulation of phospholipid biosynthesis in isolated rat hepatocytes. Effect of different substrates. J. Biol. Chem..

[81] Core LJ, Waterfall JJ, Lis JT (2008). Nascent RNA sequencing reveals widespread pausing and divergent initiation at human promoters. Science.

[82] Hetzel J, Duttke SH, Benner C, Chory J (2016). Nascent RNA sequencing reveals distinct features in plant transcription. Proc. Natl Acad. Sci. USA.

[83] Prestwood TR, Prigozhin DM, Sharar KL, Zellweger RM, Shresta S (2008). A mouse-passaged dengue virus strain with reduced affinity for heparan sulfate causes severe disease in mice by establishing increased systemic viral loads. J. Virol..

[84] Prestwood TR (2012). Trafficking and replication patterns reveal splenic macrophages as major targets of dengue virus in mice. J. Virol..

[85] Marchetto MC (2010). A model for neural development and treatment of Rett syndrome using human induced pluripotent stem cells. Cell.

[86] Zhu Z (2020). Zika virus targets glioblastoma stem cells through a SOX2-integrin alphavbeta5 Axis. Cell Stem Cell.

[87] Lanciotti RS, Lambert AJ, Holodniy M, Saavedra S, Signor Ldel C (2016). Phylogeny of Zika virus in Western Hemisphere, 2015. Emerg. Infect. Dis..

[88] Bos S (2018). The structural proteins of epidemic and historical strains of Zika virus differ in their ability to initiate viral infection in human host cells. Virology.

[89] Heang V (2012). Zika virus infection, Cambodia, 2010. Emerg. Infect. Dis..

[90] Elong Ngono A (2017). Mapping and role of the CD8(+) T cell response during primary Zika virus infection in mice. Cell Host Microbe.

[91] Clark, A. E. et al. Zika virus is transmitted in neural progenitor cells via cell-to-cell spread and infection is inhibited by the autophagy inducer trehalose. *J. Virol*. 10.1128/JVI.02024-20 (2020). 10.1128/JVI.02024-20 PMC8092816 33328307

[92] Yoon SB (2019). Real-time PCR quantification of spliced X-box binding protein 1 (XBP1) using a universal primer method. PLoS One.

[93] Troegeler A (2014). An efficient siRNA-mediated gene silencing in primary human monocytes, dendritic cells and macrophages. Immunol. Cell Biol..

[94] Heinz S (2010). Simple combinations of lineage-determining transcription factors prime cis-regulatory elements required for macrophage and B cell identities. Mol. Cell.

[95] Love MI, Huber W, Anders S (2014). Moderated estimation of fold change and dispersion for RNA-seq data with DESeq2. Genome Biol..

[96] Tripathi S (2015). Meta- and Orthogonal Integration of Influenza “OMICs” Data Defines a Role for UBR4 in Virus Budding. Cell Host Microbe.

[97] de Hoon MJ, Imoto S, Nolan J, Miyano S (2004). Open source clustering software. Bioinformatics.

[98] Saldanha AJ (2004). Java Treeview—extensible visualization of microarray data. Bioinformatics.

[99] Karlsson, M. et al. A single-cell type transcriptomics map of human tissues. *Sci. Adv.* **7**, 10.1126/sciadv.abh2169 (2021). 10.1126/sciadv.abh2169 PMC8318366 34321199

