# Supplementary material for: SREBP2-dependent lipid gene transcription enhances the infection of human dendritic cells by Zika virus
Source: Nat Commun. 2022 Sep 12;13:5341. doi: 10.1038/s41467-022-33041-1 (PMC9465152; doi:10.1038/s41467-022-33041-1)
Supplement: Supplementary file 1 — Supplementary Information [file 41467_2022_33041_MOESM1_ESM.pdf]

**Supplementary Information**

# SREBP2-Dependent Lipid Gene Transcription Enhances the Infection of Human Dendritic Cells by Zika virus

Emilie Branche[1], Sujan Shresta[1] * and Aaron F Carlin[2] *

[1] Center for Infectious Disease and Vaccine Research, La Jolla Institute for Immunology, La Jolla, CA 92037.

[2] Departments of Pathology and Medicine, School of Medicine, University of California, San Diego, La Jolla, CA 92093.

*** Corresponding Authors: Email:** sujan@lji.org (SS); acarlin@health.ucsd.edu (AFC)

## Supplementary Figures

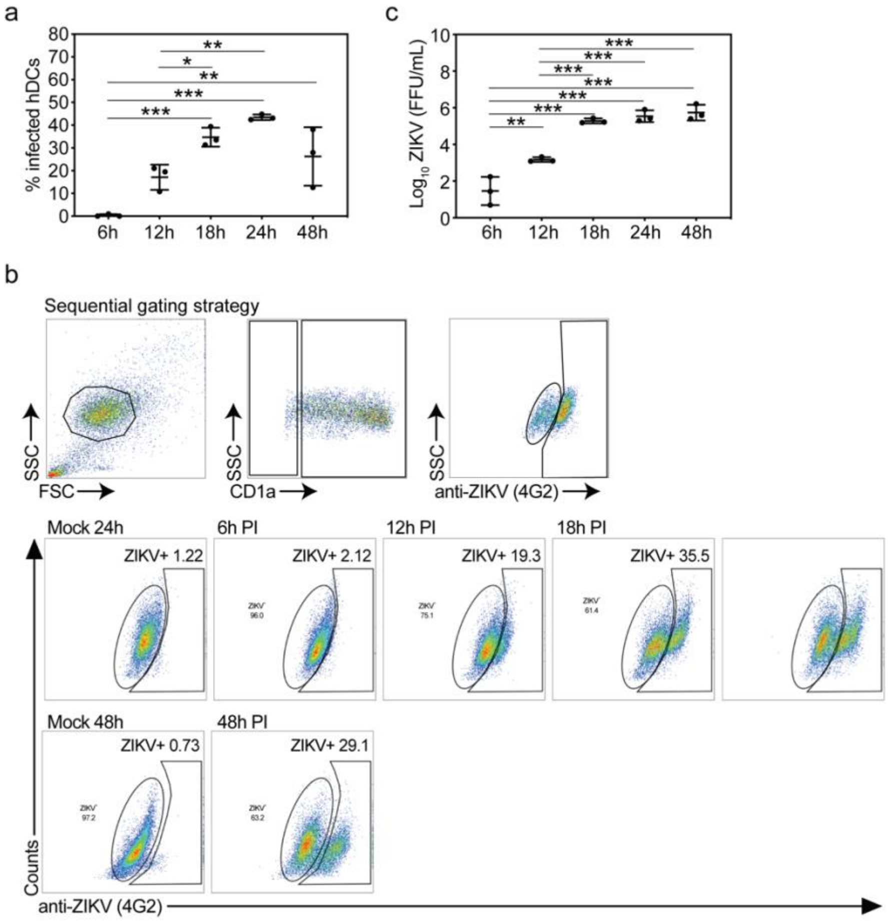


**Supplementary Figure 1: ZIKV infection of human moDCs.** Human moDCs were mock-infected or infected with ZIKV SD001 at a multiplication of infection (MOI) of 0.5. (a) Infection of cells was analyzed at the indicated times by staining with 4G2 followed by flow cytometry. (b) Sequential gating strategy used to analyze infected (4G2+) moDCs (CD1a+) for Supplementary Figures 1a, 1c and 3e. and representative flow cytometry dot plots showing gating of ZIKV-infected primary human moDCs from one donor at indicated time points post-infection (c) Focus-forming assay of secreted infectious viral particles. Data are presented as the mean ± SD. Symbols represent moDCs derived from 3 individual different donors. **P < 0.01, ***P < 0.001 by one-way ANOVA with Tukey's correction for multiple comparisons. Source data and exact P values are provided as a Source Data file.

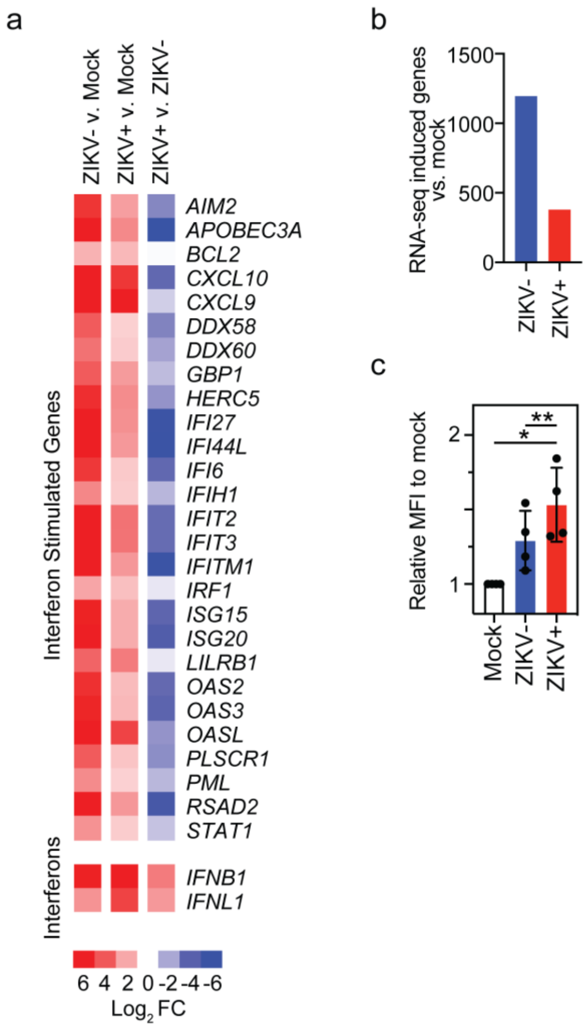


**Supplementary Figure 2: ZIKV infection suppresses expression of viral defense response genes.** Human moDCs from 4 different donors were infected for 24 h with ZIKV SD001 at a MOI 0.5 and then subjected to RNA-seq analysis. (a) Heat map of the relative expression of genes upregulated in ZIKV+ *vs* Mock moDCs within the defense response to virus GO category. (b) Number of upregulated genes in ZIKV− or ZIKV+ moDCs compared with Mock cells as determined by RNA-seq (FC >2 and FDR <0.01). (c) Neutral lipids in moDCs were stained with BODIPY® 493/503 and lipid levels were quantified by flow cytometry. Data are presented as the mean ± SD. Symbols represent moDCs derived from individual donors, n=4 biologically independent experiments. *P < 0.05, **P < 0.01 by repeated-measures one-way ANOVA with Tukey's correction for multiple comparisons. Source data and exact P values are provided as a Source Data file.

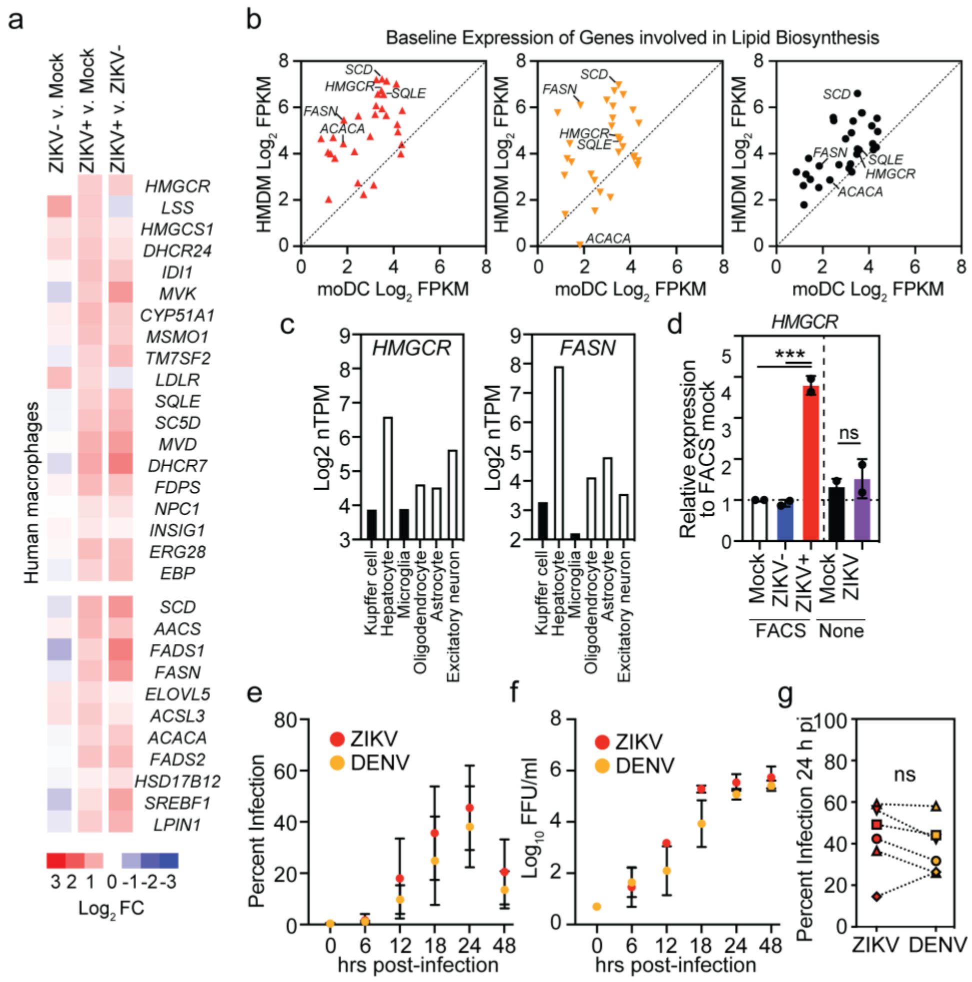


**Supplementary Figure 3: Upregulation of lipid-related gene expression in ZIKV-infected cells.** (a) Heat map of the expression of selected lipid-related genes (see also Fig. 1e), as determined by RNA-seq, in human monocyte-derived macrophages (HMDMs) infected with ZIKV FSS13025 for 18 h (MOI 1) in the presence of 0.6% (vol/vol) DENV human immune serum (antibody-mediated enhancement of infection conditions) relative to mock-infected cells as previously described[1]. (b) RNA-seq analysis of lipid biosynthetic gene expression (listed in Fig. 1e) in uninfected NPCs, Huh7.5 cells, and HMDMs relative to uninfected moDCs. (c) Single-cell expression ($Log_2$ nTPM) of *HMGCR* and *FASN* in liver and brain cells (data from The Human Protein Atlas. v21.0) [2]. (d) qRT-PCR analysis of *HMGCR* mRNA expression in ZIKV− or ZIKV+ moDCs (ZIKV BEH819015, 24 h, (MOI 1) relative to mock-infected cells. (e, f) Relative infectivity of DENV2 UIS353 (MOI 1) and ZIKV SD001 (MOI 0.5) in moDCs. Cells or supernatants were collected at the indicated times and analyzed (e) by flow cytometry after staining of virus-infected moDCs with 4G2, and (f) focus-forming assay of cell supernatants. (g) Quantification of virus-infected moDCs at 24 h post-infection by 4G2 staining followed by flow cytometry. Dashed line connects ZIKV and DENV infection of moDCs derived from the same individual. Data are presented as the mean (c) or mean ± SD (d–f) of (e) n=6 (ZIKV) or 4 (DENV) donors, (f) n=3 donors, and (g) n=6 donors. ns, Not significant, ***P < 0.001 by one-way ANOVA with Tukey's correction for multiple comparisons (d)[FACS] or two-sided unpaired t-test (d) [None],mixed-effects analysis (e), two-way ANOVA (f) with Sidak correction for multiple comparison (e,f), or two-sided paired t-test (g). Source data and exact P values are provided as a Source Data file.

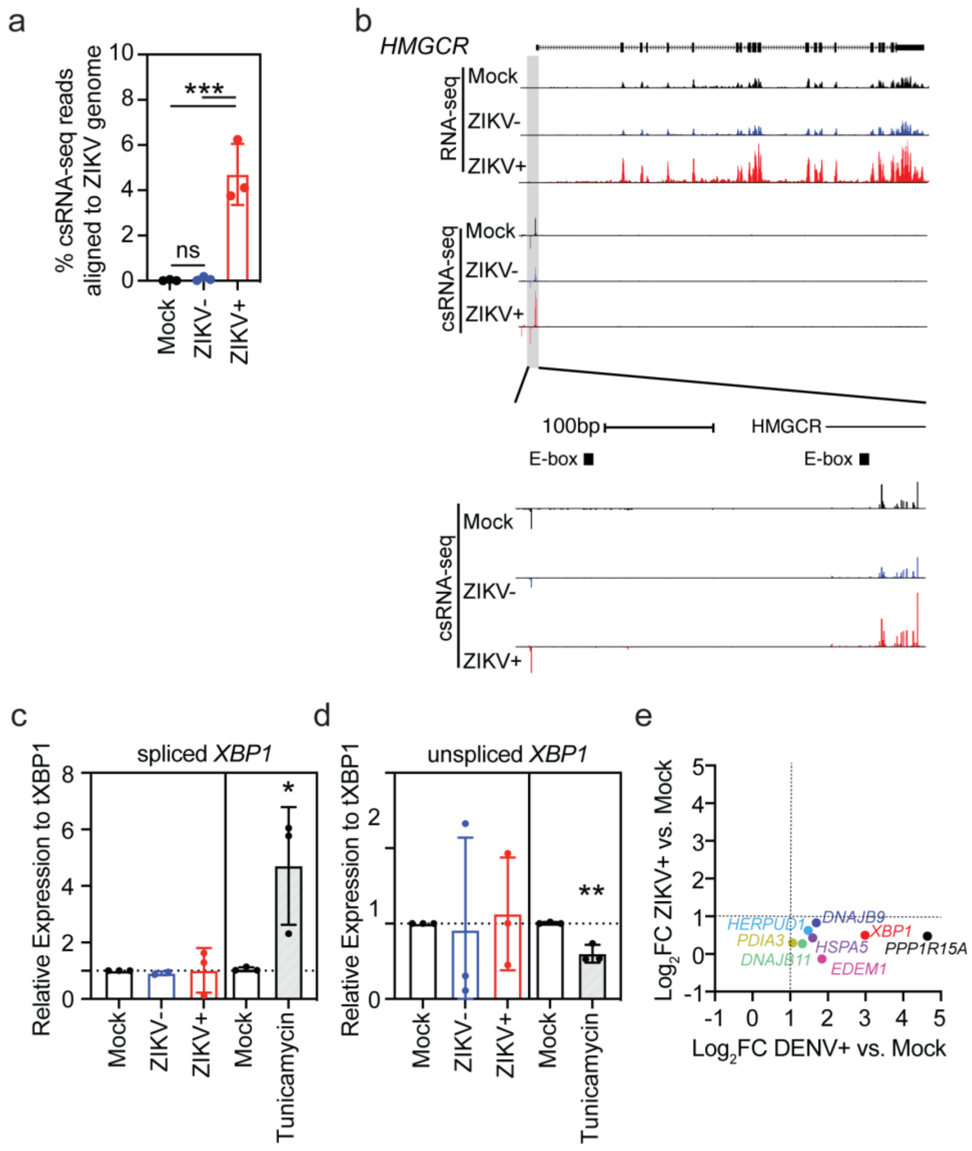


**Supplementary Figure 4: Regulation of ZIKV-induced expression of lipid-related genes**
(a) Percent csRNA-seq reads aligned to the ZIKV genome in Mock, ZIKV−, and ZIKV+ moDCs. (b) UCSC browser visualization of the *HMGCR* locus with RNA-seq, and csRNA-seq in mock, ZIKV−, and ZIKV+ moDCs. RNA-seq and csRNA-seq are strand-specific, with positive- and negative-strand transcription displayed above and below the central line, respectively. (c, d) qRT-PCR analysis of spliced (sXBP1) and unspliced (uXBP1) *XBP1* in Mock, ZIKV−, ZIKV+, or tunicamycin-treated uninfected moDCs. mRNA levels were normalized to total *XBP1* (tXBP1) levels [3]. (e) RNA-seq analysis of the expression of endoplasmic reticulum stress-associated genes in ZIKV SD001-infected or DENV2 UIS353-infected moDCs relative to mock-infected moDCs. Data are presented as the mean ± SD. Symbols represent moDCs derived from individual donors, n=3 biologically independent experiments. ns, Not significant, *P < 0.05, **P < 0.01 by one-way ANOVA with Tukey's correction for multiple comparisons (Mock *vs* ZIKV+ or ZIKV−) or t-test (Mock *vs* tunicamycin). Source data and exact P values are provided as a Source Data file.

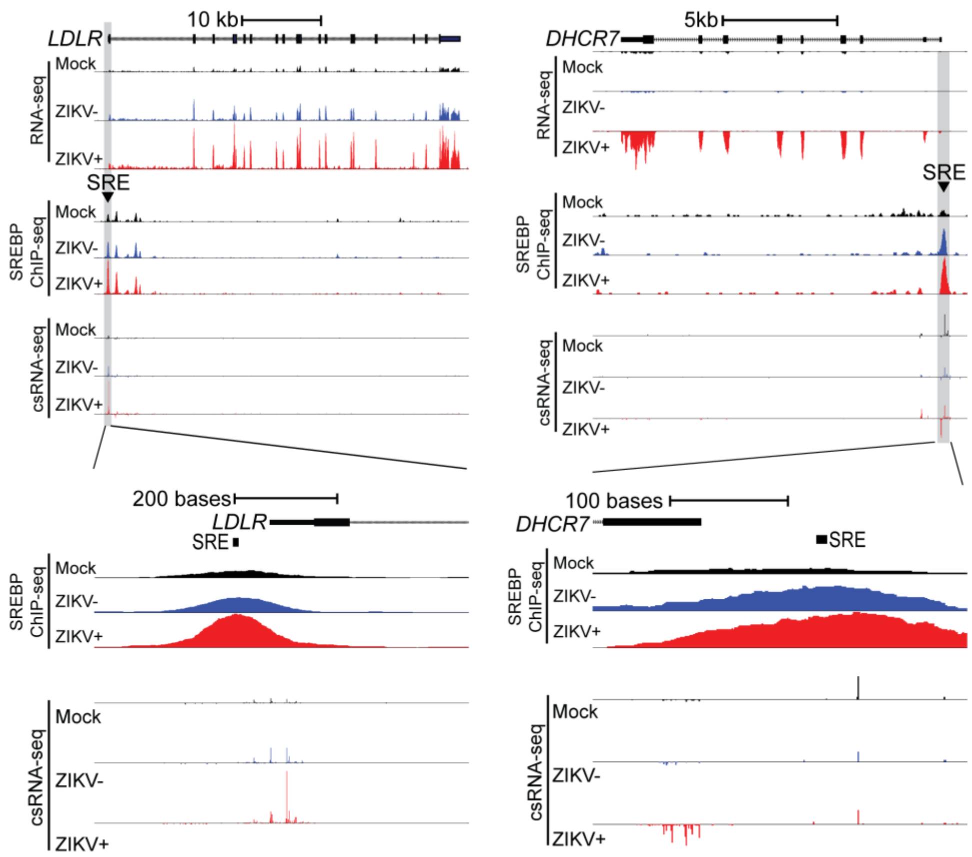


**Supplementary Figure 5: Increased SREBP recruitment to promoter SREs correlates with downstream csRNA-seq reads.** UCSC browser visualization of the *LDLR* and *DHCR7* loci with RNA-seq, SREBP ChIP-seq, and csRNA-seq in mock, ZIKV−, and ZIKV+ moDCs. RNA-seq and csRNA-seq are strand-specific, with positive- and negative-strand transcription displayed above and below the central line, respectively.

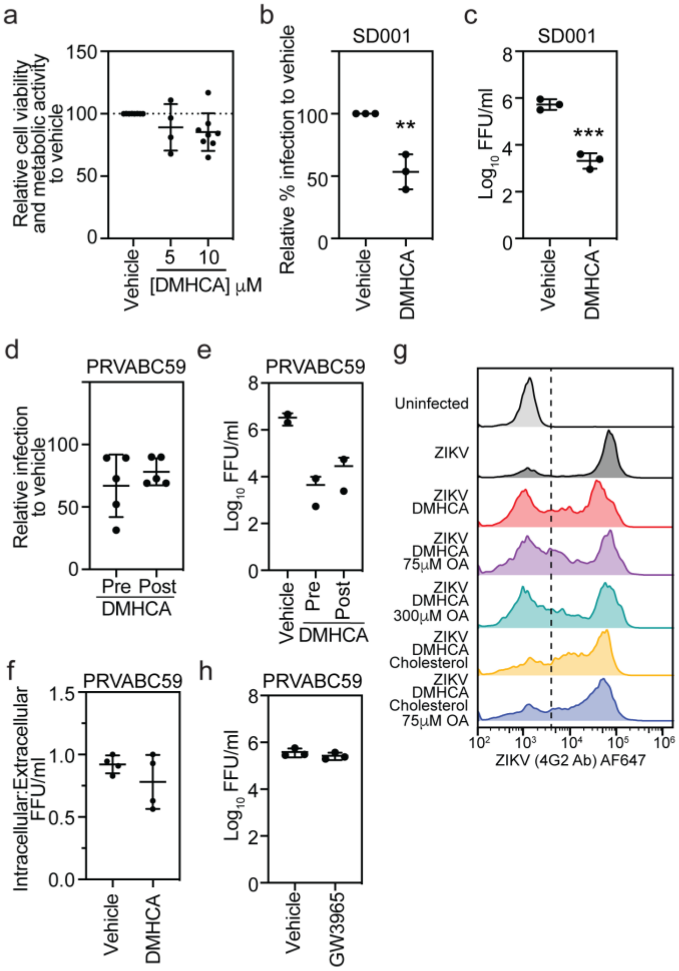


**Supplementary Figure 6: DMHCA treatment of moDCs reduces the secretion of infectious ZIKV particles.** (a) moDCs were treated with indicated with vehicle (ethanol) or 5 or 10 μM DMHCA for 28 h and metabolic activity/cell viability was measured by the MTS assay. N=8 (vehicle, 10μM) or 4 (5μM) biologically independent experiments. (b, c) moDCs were treated with vehicle or DMHCA (10μM) for 4 h and then infected with ZIKV SD001 (MOI 0.5). At 24 h post-infection (pi), (b) ZIKV+ cells were quantified by flow cytometry or (c) supernatants were assayed for infectious ZIKV particles by FFA. (d, e) Vehicle or DMHCA (10 μM) were added to moDCs at 4 h before (pre) or 2.5 h after (post) infection with ZIKV PRVABC59 (MOI 1). At 24 h pi, (d) ZIKV+ cells were quantified by flow cytometry or (e) supernatants were assayed for infectious ZIKV particles by FFA. N=5 (d) or n=2 (e) biologically independent experiments. (f) Cells were treated as for B and C and intracellular ZIKV infectious particles was quantified by FFA at 24 h pi. N=4 biologically independent experiments. (g) Flow cytometry histograms of uninfected moDCs or ZIKV+ moDCs treated as described for figure 5f with vehicle or 10 μM DMHCA with or without 75 or 300 μM oleic acid–BSA (OA) or 25μg/ml cholesterol–methyl-β-cyclodextrin (Cholesterol). Cells were analyzed at 24 h pi. Data from one representative donor are shown. (h) Human moDCs were infected with ZIKV PRVABC59 (MOI 1) and GW3965 (1 μM) was added at 2.5 h pi. At 24 h pi, supernatants were collected and infectious particles were quantified by FFA. Data are presented as the mean ± SD. Symbols represent moDCs derived from individual donors. N=3 (b), (c) and (h) biologically independent experiments. **P < 0.01, ***P < 0.001 by one-way ANOVA with Tukey's correction for multiple comparisons. Source data and exact P values are provided as a Source Data file.

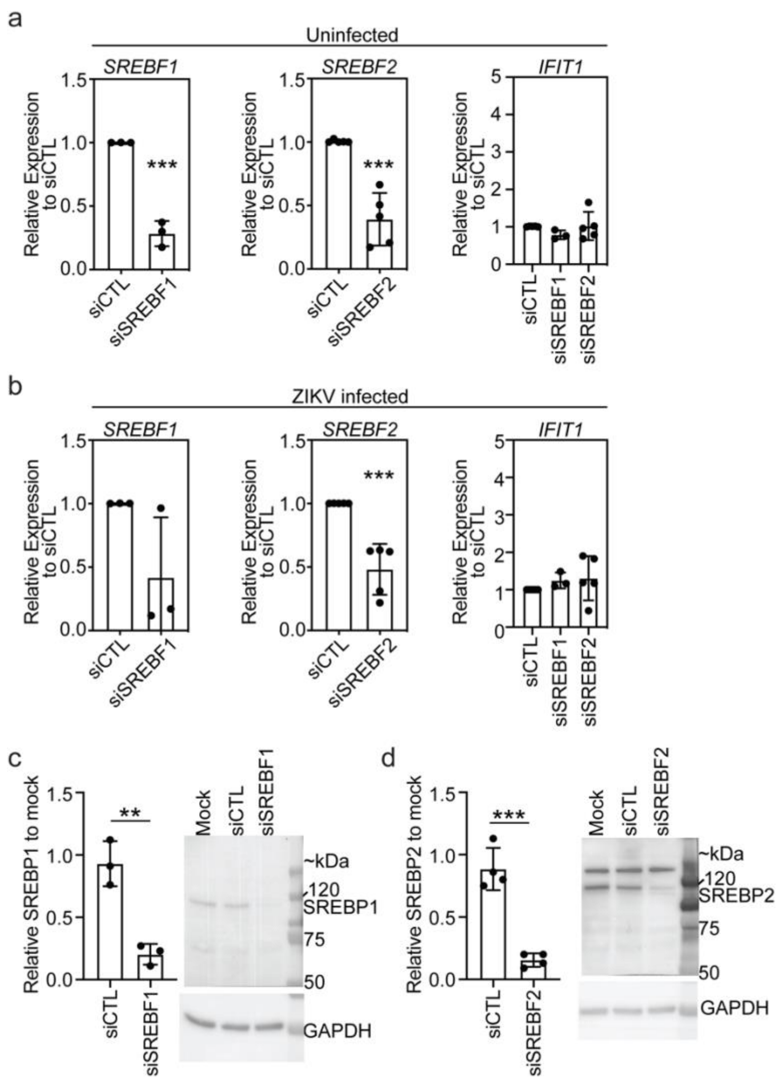


**Supplementary Figure 7: Characterization of uninfected and ZIKV-infected moDCs after *SREBF1* or *SREBF2* knockdown.** (a–d) Human moDCs were transfected with a control siRNA (siCTRL), si*SREBF1*, or si*SREBF2* for 24 h and then infected with ZIKV SD001 (MOI 0.5) for an additional 24 h. (a, b) qRT-PCR analysis of *SREBF1, SREBF2, and IFIT1* mRNAs in (a) mock-infected or (b) ZIKV-infected cells. N=5 (siCTL, siSREBF2) or n=3 (siSREBF1) biologically independent experiments. (c, d) Western blot analysis of (c) SREBP1 or (d) SREBP2 proteins in mock- or ZIKV-infected moDCs. Western blots and bar charts show data from one representative donor and n=4 (siSREBF2) or n=3 (siSREBF1) biologically independent experiments, respectively. Data are presented as the mean ± SD. Symbols represent moDCs derived from individual donors. **P < 0.01, *** P < 0.001 by unpaired two-sided t-test except for *IFIT1* mRNA, which was assessed by ANOVA with Tukey's correction for multiple comparisons. Source data and exact P values are provided as a Source Data file.

Supplementary Table 1: Antibodies used in the study

| Antibody | Company | Catalog number | Antibody dilution or amount |
|---|---|---|---|
| Peroxidase AffiniPure goat anti-mouse IgG, F(ab′)$_2$ fragment-specific | Jackson ImmunoResearch | 115-035-072 | 1:1000 FFA |
| 4G2 mouse monoclonal Clone D1-4G2-4-14, hybridoma from ATCC | BIOXCELL | Lot# 630816D1 | 1:100 FC, 1μg/ml FFA |
| SREBP1 rabbit polyclonal | Santa Cruz Biotechnology | sc-8984X | 2μg per ChIP |
| SREBP1 rabbit polyclonal | Thermo Fisher Scientific | PA1-337 | 2μg per ChIP |
| SREBP2 goat polyclonal | R&D Systems | AF7119 | 2μg per ChIP; 1:1000 WB |
| SREBP-1 rabbit monoclonal | Sigma-Aldrich | MABS1987 | 1:1000 WB |
| GAPDH Rabbit monoclonal D16H11 | Cell Signaling Technology | 5174S | 1:1000 WB |
| Goat Anti-Rabbit, Polyclonal Immunoglobulins, HRP | Agilent | P044801-2 | 1:10,000 WB |
| Rabbit Anti-Goat, IgG HRP-conjugated Antibody | R&D systems | HAF017 | 1:10,000 WB |

Flow Cytometry (FC), Western blot (WB); ChIP (ChIP-seq), Focus Forming Assay (FFA)

Supplementary Table 2: siRNA used in the study

| siRNA | Company | Catalog number |
|---|---|---|
| SMARTpool ON-TARGETplus SREBF1 siRNA | Dharmacon | L-006891-00-0005 |
| SMARTpool ON-TARGETplus SREBF2 siRNA | Dharmacon | L-009549-00-0005 |
| SMARTpool ON-TARGETplus FASN siRNA | Dharmacon | L-003954-00-0005 |
| ON-TARGETplus Non-targeting Pool | Dharmacon | D-001810-10-05 |

Supplementary Table 3: Compounds used in the study

| **Drugs/treatments** | **Company** | **Catalog number** |
|---|---|---|
| DMHCA | Avanti Polar Lipids | 700125 |
| GW3965 | Millipore Sigma | G6295 |
| Cholesterol–methyl-β-cyclodextrin (water soluble) | Millipore Sigma | C4951 |
| Oleic acid–BSA | Millipore Sigma | O3008 |

Supplementary Table 4: Primers used in the study

| **qPCR Primers** | **Forward** | **Reverse** |
|---|---|---|
| *RPLP0* | 5′-GTGTTCGACAATGGCAGCAT-3′ | 5′-GACACCCTCCAGGGAGCGA-3′ |
| *IFIT1* | 5′-GCCTCCTTGGGTTCGTCTAC-3 | 5′- AAGTCAGCAGCCAGTCTCAG-3′ |
| *FASN* | 5′-ACAGCGGGGAATGGGTACT-3′ | 5′-GACTGGTACAACGAGCGGAT-3′ |
| *DHCR7* | 5′-GCTGCAAAATCGCAACCCAA-3′ | 5′-GCTCGCCAGTGAAAACCAGT-3′ |
| *SCD* | 5′-TCTAGCTCCTATACCACCACCA-3′ | 5′-TCGTCTCCAACTTATCTCCTCC-3′ |
| *HMGCR* | 5′-TGATTGACCTTTCCAGAGCAA-3′ | 5′-CTAAAATTGCCATTCCACGAG-3′ |
| ZIKV | 5′-TTGGTCATGATACTGCTGATTGC-3′ | 5′-CCTTCCACAAAGTCCCTATTGC-3′ |
| *SREBF1* | 5′-ACAGTGACTTCCCTGGCCTAT-3′ | 5′-GCATGGACGGGTACATCTTCAA-3′ |
| *SREBF2* | 5′-AACGGTCATTCACCCAGGTC-3′ | 5′-GGCTGAAGAATAGGAGTTGCC-3′ |
| *uXBP1* | 5’-CAGACTACGTGCACCTCTGC-3’ | 5’-CTGGGTCCAAGTTGTCCAGAAT-3’ |
| *sXBP1* | 5’-GCTGAGTCCGCAGCAGGT-3’ | 5’-CTGGGTCCAAGTTGTCCAGAAT-3’ |
| *tXBP1* | 5’-TGAAAAACAGAGTAGCAGCTCAGA-3’ | 5’-CCCAAGCGCTGTCTTAACTC-3' |

Supplementary Table 5: Key reagents used in the study

| **Key Reagents** | **Company** | **Catalog number** |
|---|---|---|
| Histopaque 1077 | M&P Biomedical | 190837 |
| Molecular grade water | Corning | 46-000-CM |
| MEM-α | Gibco | 12561-056 |
| RPMI + GlutaMAX | Gibco | 61870-036 |
| HEPES | Life Technologies, GIBCO | BP299100 |
| Penicillin/streptomycin | Life Technologies, GIBCO | 15-140-163 |
| Pan Monocyte Isolation Kit, human | Miltenyi Biotech | 130-096-537 |
| Recombinant human GM-CSF | Pepro Tech | 300-03 |
| Recombinant human IL-4 | Pepro Tech | 200-04 |
| LS columns | Miltenyi Biotech | 130-042-401 |
| BD Cytofix/Cytoperm | BD Biosciences | 554722 |
| Paraformaldehyde 16% | Electron Microscopy Sciences | 15710-S |
| Saponin | Sigma Aldrich | 47036-50G-F |
| Bovine serum albumin | Sigma Aldrich | A3294-100G |
| RNasin Ribonuclease Inhibitor (RNasin Plus) | Promega | N2615 |
| EDTA (0.5 M), pH 8.0, RNase-free | Life Technologies | SF100-4 |
| Zombie violet fixable viability kit | BioLegend | 423113 |
| PermWash concentrate | BD Biosciences | 51-2091KZ |
| Human FcX True stain (Fc receptor blocking solution) | BioLegend | 422302 |
| CMC sodium salt, medium viscosity | Sigma Aldrich | C9481-500G |
| Triton X-100 | Sigma Aldrich | 9002-93-1 |
| TruBlue | SERA CARE | 5510-0030 |
| 10% Buffered formalin phosphate | FISCHER CHEMICAL | SF100-4 |
| Alexa Fluor 647 labeling kit | Invitrogen | A20186 |
| CellTiter 96 Aqueous One Solution Cell Proliferation Assay System | Promega | G3582 |
| StemFect RNA transfection kit | Reprocell | 00-0069 |
| Dynabeads, Protein A | Thermo Fisher Scientific | 10002D |
| Dynabeads, Protein G | Thermo Fisher Scientific | 10004D |

## Supplementary References


1 Carlin, A. F. *et al.* Deconvolution of pro- and antiviral genomic responses in Zika virus-infected and bystander macrophages. *Proc Natl Acad Sci U S A* **115**, E9172-E9181, doi:10.1073/pnas.1807690115 (2018).

2 Karlsson, M. *et al.* A single-cell type transcriptomics map of human tissues. *Sci Adv* **7**, doi:10.1126/sciadv.abh2169 (2021).

3 Yoon, S. B. *et al.* Real-time PCR quantification of spliced X-box binding protein 1 (XBP1) using a universal primer method. *PLoS One* **14**, e0219978, doi:10.1371/journal.pone.0219978 (2019).
