# Supplementary material for: SREBP2-dependent lipid gene transcription enhances the infection of human dendritic cells by Zika virus
Source: Nat Commun. 2022 Sep 12;13:5341. doi: 10.1038/s41467-022-33041-1 (PMC9465152; doi:10.1038/s41467-022-33041-1)
Supplement: Supplementary file 2 — Reporting Summary [file 41467_2022_33041_MOESM2_ESM.pdf]

nature portfolio

Corresponding author(s): Aaron Carlin
Sujan Shresta

Last updated by author(s): Apr 18, 2022

Reporting Summary

Nature Portfolio wishes to improve the reproducibility of the work that we publish. This form provides structure for consistency and transparency in reporting. For further information on Nature Portfolio policies, see our Editorial Policies and the Editorial Policy Checklist.

Statistics

For all statistical analyses, confirm that the following items are present in the figure legend, table legend, main text, or Methods section.

n/a	Confirmed	
☐	☒	The exact sample size (*n*) for each experimental group/condition, given as a discrete number and unit of measurement
☐	☒	A statement on whether measurements were taken from distinct samples or whether the same sample was measured repeatedly
☐	☒	The statistical test(s) used AND whether they are one- or two-sided<br>*Only common tests should be described solely by name; describe more complex techniques in the Methods section.*
☐	☒	A description of all covariates tested
☐	☒	A description of any assumptions or corrections, such as tests of normality and adjustment for multiple comparisons
☐	☒	A full description of the statistical parameters including central tendency (e.g. means) or other basic estimates (e.g. regression coefficient) AND variation (e.g. standard deviation) or associated estimates of uncertainty (e.g. confidence intervals)
☐	☒	For null hypothesis testing, the test statistic (e.g. *F*, *t*, *r*) with confidence intervals, effect sizes, degrees of freedom and *P* value noted<br>*Give P values as exact values whenever suitable.*
☒	☐	For Bayesian analysis, information on the choice of priors and Markov chain Monte Carlo settings
☒	☐	For hierarchical and complex designs, identification of the appropriate level for tests and full reporting of outcomes
☒	☐	Estimates of effect sizes (e.g. Cohen's *d*, Pearson's *r*), indicating how they were calculated

Our web collection on statistics for biologists contains articles on many of the points above.

Software and code

Policy information about availability of computer code

Data collection	none
Data analysis	Code used to analyze data in this manuscript has been integrated into HOMER (http://homer.ucsd.edu/).<br>Additional software tools used in the study including STAR 2.7.1a, Cluster3.0, Java TreeView, R/Biocondutor(DESeq2), Excel, FlowJo 10.8, Metascape, UCSC browser, and Prism Graphpad 8.4.3.

For manuscripts utilizing custom algorithms or software that are central to the research but not yet described in published literature, software must be made available to editors and reviewers. We strongly encourage code deposition in a community repository (e.g. GitHub). See the Nature Portfolio guidelines for submitting code & software for further information.

Data

Policy information about availability of data

All manuscripts must include a data availability statement. This statement should provide the following information, where applicable:

- Accession codes, unique identifiers, or web links for publicly available datasets
- A description of any restrictions on data availability
- For clinical datasets or third party data, please ensure that the statement adheres to our policy

All human RNA-seq, csRNA-seq, and ChIP-seq data described in this manuscript will be available at the National Center for Biotechnology Information Gene Expression Omnibus (GEO) study GSE161783 and GSE118305.

Field-specific reporting

Please select the one below that is the best fit for your research. If you are not sure, read the appropriate sections before making your selection.

- [x] Life sciences
- [ ] Behavioural & social sciences
- [ ] Ecological, evolutionary & environmental sciences

For a reference copy of the document with all sections, see nature.com/documents/nr-reporting-summary-flat.pdf

Life sciences study design

All studies must disclose on these points even when the disclosure is negative.

Sample size	Our published results and preliminary data demonstrate that we can identify hundreds to thousands of differentially regulated transcripts, regulatory elements, or TF binding sites (fold change > 2, FDR < 5%) using 3 replicates per genomics assay. This suggests that 3 replicates should be sufficiently powered to identify similar numbers of features in the experiments proposed in this study.
Data exclusions	No data was excluded.
Replication	Data was repeated in at least 2 independent experiments using moDCs derived from multiple donors. Please see figure legends for the number of replicates performed for each individual experiment.
Randomization	moDCs were derived from healthy adult human volunteers. moDCs from each participant was used to generate data for virus+, virus- and mock samples. The donors were randomly selected from male and female participants between 18 and 70 years of age.
Blinding	Investigators are blinded to participant information, including demographic data, including age and sex.

Reporting for specific materials, systems and methods

We require information from authors about some types of materials, experimental systems and methods used in many studies. Here, indicate whether each material, system or method listed is relevant to your study. If you are not sure if a list item applies to your research, read the appropriate section before selecting a response.

Materials & experimental systems

n/a	Involved in the study	
[ ]	[x]	Antibodies
[ ]	[x]	Eukaryotic cell lines
[x]	[ ]	Palaeontology and archaeology
[x]	[ ]	Animals and other organisms
[ ]	[x]	Human research participants
[x]	[ ]	Clinical data
[x]	[ ]	Dual use research of concern

Methods

n/a	Involved in the study	
[ ]	[x]	ChIP-seq
[ ]	[x]	Flow cytometry
[x]	[ ]	MRI-based neuroimaging

Antibodies

Antibodies used	All antibodies utilized are described in Supplementary Table S1
Validation	All antibodies utilized are commercially available. Validation information is available at the manufacturer websites. All antibodies listed (Supplementary Table S1) performed as expected.Per manufacturer websites:115-035-072 - Polyclonal Goat anti-Mouse IgG, F(ab')$_2$ fragment specific conjugated to horseradish peroxidase. 5174S GAPDH (D16H11)XP® Rabbit mAb detects endogenous levels of total GAPDH protein in Human, Mouse, Rat, Monkey. It has been used in western blot, IHC and IF. sc-8984X Detects SREBP-1 epitope corresponding to amino acids 41-200 mapping near the N-terminus of SREBP-1 of human origin. Can be used for western blotting, IP, IF, IHC. PA1-337 detects SREBP-1 in mouse and rat samples in Western blot and ICC/IF procedures. The immunogen is identical in human and rat. AF7199 - Detects human SREBP-2 and has been used in ELISA, Western blots, ChIP, IF. MABS1987 - Anti-SREBP-1 Antibody, clone 20B12 detects human SREBP1 in western blot. P044801-2 - Polyclonal Goat Anti-Rabbit Immunoglobulins/HRP. ELISA, affinity-isolated secondary antibody conjugated with HRP. HAF017 - Polyclonal Rabbit Anti-Goat Immunoglobulin/HRP. Detects goat IgG heavy and light chains in direct ELISAs and western blots.

Eukaryotic cell lines

Policy information about cell lines origin.

Cell line source(s)	moDCs were derived from human blood. The source of BHK21 and C6/36 are from ATCC and Huh7.5 are from Apath LLC. Thehuman induced pluripotent cell line WT-126 was derived in the lab of Alysson Muotri. This line was obtained from Dr. Muotri by Dr. Jeremy Rich.
Authentication	Primary human monocytes were isolated from human blood and differented in IL-4 and GM-CSF to produce moDCs. moDC differentiation was monitored by staining for CD14 and CD1A. The human induced pluripotent cell line WT-126 verified by IF staining at multiple stages of differentiation and authenticated by morphology and STR profiling. Cell lines were obtained from reputable vendors, ATCC or APATH LLC, verified by microscopic inspection and utilized at low passage numbers.
Mycoplasma contamination	All cell lines tested negative for mycoplasma contamination.
Commonly misidentified lines (See ICLAC register)	None

Human research participants

Policy information about studies involving human research participants

Population characteristics	Human blood was obtained from healthy adult volunteers and was deidentified under Protocol VD-057-0217 (La Jolla Institute) or Protocol 181624 (UCSD). Participant pools consist of healthy males and females between the ages of 18-70.
Recruitment	Healthy adult volunteers were recruited to donate blood for research purposes.Posters are placed in common areas throughout LJI, and UCSD campus. As well as and bulk e-mails are sent out to recruit potential donors.
Ethics oversight	Blood was obtained from consented healthy participants under VD-057-0217, and the UCSD IRB, Protocol 181624. All recruited volunteers provided written informed consent. Human blood was obtained from healthy volunteers and deidentified prior to processing.

ChIP-seq

Data deposition

☒ Confirm that both raw and final processed data have been deposited in a public database such as GEO.

☒ Confirm that you have deposited or provided access to graph files (e.g. BED files) for the called peaks.

Data access links<br>*May remain private before publication.*	National Center for Biotechnology Information Gene Expression Omnibus (GEO) study GSE161783 and GSE118305.
Files in database submission	All human RNA-seq, csRNA-seq, and ChIP-seq data described in this manuscript
Genome browser session<br>(e.g. UCSC)	https://genome.ucsc.edu/s/acarlin/ZIKV_SREBP

Methodology

Replicates	ChIP-seq was performed on moDCs derived from 3 donors.
Sequencing depth	Samples were sequenced using the Illumina NextSeq 500 platform using 51 cycles (ChIP-seq) single-end sequencing per manufacturer instructions. Approximately 15 million reads for Mock, ZIKV- and ZIKV+.
Antibodies	Anti-SREBP Abs (Santa Cruz Biotechnology sc-8984X, Thermo Fisher Scientific PA1-337, R&D Systems AF7119) were used and described in Supplementary Table S1 and SREBP ChIP-seq methods section.
Peak calling parameters	SREBP ChIP-seq peaks were called using tags from three replicates per group with respective input DNA as background using HOMER's getDifferentialPeaksReplicates.pl using the "-style factor" (5-fold change cutoff over background and FDR <0.001 for peak identification)
Data quality	Peaks were called based on 5-fold enrichment over background and FDR <0.001. Peaks from each group (Mock, ZIKV-, ZIKV+) were merged and identified 16800 peaks total.
Software	Homer v4 was used for the ChIP-seq analysis.

Flow Cytometry

Plots

Confirm that:

☒ The axis labels state the marker and fluorochrome used (e.g. CD4-FITC).

☒ The axis scales are clearly visible. Include numbers along axes only for bottom left plot of group (a 'group' is an analysis of identical markers).

☒ All plots are contour plots with outliers or pseudocolor plots.

☒ A numerical value for number of cells or percentage (with statistics) is provided.

Methodology

Sample preparation	Following viral infection, moDCs were collected, stained with Zombie Violet™ Fixable Viability stain (BioLegend), washed, fixed and permeabilized using BD Cytofix/Cytoperm reagents, and then intracellularly stained with FITC- or AF647-conjugated 4G2 mAb. Cells were washed twice with BD Perm/Wash Buffer and resuspended in FACS buffer.
Instrument	Flow cytometry was performed on an LSRII flow cytometer (BD Biosciences) or MA900 cell sorter (Sony)
Software	Flow cytometry was analyzed using Flowjo version 10
Cell population abundance	Cell population abundance is contained in Supp Figure 1a, 1b and Supp Figure 3e. Sample purity of infection was determined by RNA-seq using reads mapped to the viral genome.

Gating strategy

Sequentia1 gating strategy is in Supp Figure 1b. Ce11s were gated on FSC/SSC then CD1a then 4G2 (antif1avivirus enve1ope)

☒ Tick this box to confirm that a figure exemplifying the gating strategy is provided in the Supplementary Information.
